# Supplementary material for: Design of facilitated dissociation enables timing of cytokine signalling
Source: Nature. 2025 Sep 24;647(8089):528–35. doi: 10.1038/s41586-025-09549-z (PMC12611780; doi:10.1038/s41586-025-09549-z)
Supplement: Supplementary file 1 — Supplementary Notes 1–5, providing additional description and analysis of the designs; Supplementary Figs. 1–17, depicting design and experimental strategies and additional data; and Supplementary Tables 1–4, listing fit parameters for kinetics and DEER data and providing sequences for all designs. [file 41586_2025_9549_MOESM1_ESM.pdf]

nature portfolio

https://doi.org/10.1038/s41586-025-09549-z

Supplementary information

Design of facilitated dissociation enables timing of cytokine signalling

In the format provided by the authors and unedited

Design of facilitated dissociation enables timing of cytokine signaling

Supplementary Information

Adam J. Broerman[*1,2,3], Christoph Pollmann[4], Yang Zhao[5], Mauriz A. Lichtenstein[1,6], Mark D. Jackson[7], Maxx H. Tessmer[7], Won Hee Ryu[8], Masato Ogishi[5], Mohamad H. Abedi[1,2], Danny D. Sahtoe[1,2], Aza Allen[1,2], Alex Kang[1,2], Joshmyn De La Cruz[1,2], Evans Brackenbrough[1,2], Banumathi Sankaran[9], Asim K. Bera[1,2], Daniel M. Zuckerman[8], Stefan Stoll[7], K. Christopher Garcia[5,10], Florian Praetorius[*†1,2], Jacob Piehler[4], David Baker[*1,2,11]

1. Institute for Protein Design, University of Washington, Seattle, WA, USA.
2. Department of Biochemistry, University of Washington, Seattle, WA, USA.
3. Department of Chemical Engineering, University of Washington, Seattle, WA, USA.
4. Department of Biology/Chemistry and Center for Cellular Nanoanalytics, Osnabrück University, Osnabrück, Germany.
5. Departments of Molecular and Cellular Physiology and Structural Biology, Stanford University School of Medicine, Stanford, CA, USA.
6. Institute for Chemistry and Biochemistry, Freie Universität Berlin, Berlin, Germany.
7. Department of Chemistry, University of Washington, Seattle, WA, USA.
8. Department of Biomedical Engineering, Oregon Health and Science University, Portland, OR, USA.
9. Molecular Biophysics and Integrated Bioimaging, Lawrence Berkeley National Laboratory, Berkeley, CA, USA.
10. Howard Hughes Medical Institute, Stanford University, Stanford, CA, USA.
11. Howard Hughes Medical Institute, University of Washington, Seattle, WA, USA.

* Corresponding author email: broerman@uw.edu, florian.praetorius@ist.ac.at, dabaker@uw.edu

† Current address: Institute of Science and Technology Austria, Klosterneuburg, Austria

Table of Contents

Supplementary Note 1 | Nomenclature 3

Supplementary Note 2 | Sequence features of the register-shift switches 3

Supplementary Note 3 | Dynamics within the target·AS1 and target·AS1·effector complexes 3

Supplementary Note 4 | Strain geometry effects on facilitated dissociation 4

Supplementary Note 5 | ASNeo2 variants 4

Supplementary Figure 1 | Relationship between affinity and exchange rate 6

Supplementary Figure 2 | Dependence of facilitated dissociation on the energy of the ternary intermediate 7

Supplementary Figure 3 | Design and characterization of the target 8

Supplementary Figure 4 | Approach to designing facilitated dissociation systems 9

Supplementary Figure 5 | Negative allosteric coupling between target and effector 10

Supplementary Figure 6 | Initial characterization of register-shift host designs 11

Supplementary Figure 7 | SPR characterization of facilitated dissociation in host designs 12

Supplementary Figure 8 | Fitting SPR facilitated dissociation data 14

Supplementary Figure 9 | Stability of the AS1 and AS114 ternary complexes at high concentration 16

Supplementary Figure 10 | Modeling strain energy in the ternary complex 17

Sources of Error in this Analysis 19

Supplementary Figure 11 | Characterization of AS1 variants 22

Supplementary Figure 12 | Reverse facilitated dissociation with selected AS1 variants 23

Supplementary Figure 13 | Characterization of initial switchable IL-2 mimic designs 24

Supplementary Figure 14 | Differences in gene expression between no, transient, and sustained stimulation 25

Supplementary Figure 15 | Variation in gene sets following different stimulation conditions 27

Supplementary Figure 16 | Additional kinetic characterization of ASNeo2 and variants 28

Supplementary Figure 17 | Flow cytometry gating 29

Supplementary Table 1 | Kinetic parameters from forward and reverse facilitated dissociation experiments with target and peptide effector 30

Supplementary Table 2 | Kinetic parameters from facilitated dissociation with 3hb effector 32

Supplementary Table 3 | DEER experimental and fit parameters 33

Supplementary Table 4 | Sequences of designed proteins. 34

References 43

Supplementary Note 1 | Nomenclature

"Host" refers to the switch-binder fusion with allosterically coupled target and effector binding sites.

"Target" refers to the protein which binds tightly to the host but can be rapidly induced to dissociate.

"Effector" refers to the protein/peptide which binds to the host at a different site to cause a conformational change in the host which destabilizes target binding.

"Binder" refers to the component of the host designed to bind to the target.

"Switch" refers specifically to the effector-responsive conformational switch component of the host proteins, and we avoid using "switch" to refer to the entire host protein. This way, we distinguish between designing switches (multi-state design) and designing hosts (fusing switches to binders).

Supplementary Note 2 | Sequence features of the register-shift switches

The crystal structures of AS1 and AS5 show sequence features present in almost all designs (insets in Fig. 3a,b and Extended Data Fig. 1a,b): a positively charged residue (K60) holds the cleft open in state X and moves to interact with the negative dipole of the effector helix in state Y, and the interface between domains in the designed one-heptad register shift contains sequence motifs repeated a heptad apart (on the C-terminal domain, L84 in state X and L91 in state Y pack against the same location on the N-terminal domain).

Supplementary Note 3 | Dynamics within the target·AS1 and target·AS1·effector complexes

We solved structures of AS1 bound to the target from two different crystals, each containing two copies of the complex. Both copies in one crystal and one copy in the other closely match the state X design model with slight fluctuation in the target binding conformation (Fig. 3c, top; Extended Data Fig. 1d), whereas in the remaining copy, AS1 adopts a different conformation in which the effector-binding cleft has collapsed to resemble the closed state X of the original hinge switch (Fig. 3c, bottom). The new state X of AS1 still slightly clashes with the target (Extended Data Fig. 1c), likely causing this dynamic target binding and cleft collapse.

We explored the dynamics within the AS1 ternary complex using molecular dynamics (MD) simulations and double electron-electron resonance (DEER) spectroscopy to measure pairwise distance distributions between residues on opposing sides of the conformational switch. For the three low-energy states, the AS1 alone, target·AS1 complex, and AS1·effector complex DEER distance distributions are consistent with the distances between spin labels simulated based on the corresponding crystal structures (Extended Data Fig. 2). For the higher-energy ternary complex, the DEER distance distribution contained two peaks: one aligned with the distances simulated from the ternary complex crystal structure and from two MD trajectories started from the

AlphaFold2 (AF2) prediction of the ternary complex, and the other aligned with the distance simulated from a third trajectory (Extended Data Fig. 3). Thus, to resolve strain, the ternary complex can likely adopt multiple conformations with varying amounts of switch deformation, target deformation, and unfolding of the clashing region of the target (Extended Data Fig. 3).

Supplementary Note 4 | Strain geometry effects on facilitated dissociation

Surprisingly, the AS1 variants with the largest deformations in the strained ternary complex do not have the fastest dissociation rates: for example the predicted deformation magnitude for variant AS114 is double that of the fastest variant AS117 (Supplementary Fig. 10b,c), yet its accelerated target dissociation is 40-fold slower (Fig. 4b). This could reflect the strain being so great in state Y that the AS114 ternary complex instead occupies state X, but DEER measurements of the AS114 ternary complex confirm that it primarily occupies state Y (Extended Data Fig. 2c). Rather, designs like AS1 and AS117 must deform in directions of higher stiffness than the slower but more highly deforming variants like AS114. Due to differences in the relative switch and binder positions, the binder:target interface in the predicted ternary complex experiences shear strain in designs like AS1 and AS117 but bending strain in designs like AS114 (Supplementary Fig. 10b). Beta sheets are stiffest against shear[1,2], so deforming in a direction that shears the binder:target interface likely leads to the higher-energy ternary complexes seen in designs like AS1 and AS117. Thus, the global strain energy of the ternary complex depends on both the magnitude and direction of the deformation (Supplementary Fig. 10e).

To explore whether these designs vary not only the global strain energy of the ternary complex but also the distribution of this strain throughout the structure, we used a fluorescence polarization competition assay to measure target-induced acceleration of effector dissociation (the reverse direction of facilitated dissociation) (Fig. 4b, Supplementary Fig. 12). Designs like AS114 showed approximately equal fold acceleration in the forward and reverse directions, whereas designs like AS1 and AS117 showed 5–15-fold greater acceleration in the forward direction (Extended Data Fig. 4). This suggests that in these designs, much of the strain energy is directed towards shearing the target interface, promoting target dissociation over effector dissociation, whereas other deformation directions more uniformly distribute strain across the complex. This is supported by the crystal structures of AS1, in which the binder:target interface deforms from its binary complex conformation much more than the switch:effector interface (1.4 Å vs 0.5 Å Cα RMSD) (Extended Data Fig. 4d).

Supplementary Note 5 | ASNeo2 variants

For downstream applications, it would be desirable to have versions of ASNeo2 that 1) rapidly dissociate from IL-2Rβ after γ_c dissociates, 2) do not release active cytokine in the event of limited proteolysis, and 3) are effective at lower effector concentrations. For (1), we found that mutation H198E increases the ASNeo2:IL-2Rβ off-rate by 5-fold (Supplementary Fig. 16a). For (2), splitting the Neo-2 across the switch would ensure that activity is lost if the switch regulating it

degrades, and for (3), ASNeo2 binds the effector considerably slower than AS1 (Supplementary Fig. 16b), suggesting the effector-binding cleft is predominantly collapsed prior to effector binding and requiring higher effector concentrations than AS1 for a rapid response (Fig. 4a, 5d). To address both (2) and (3), we used a split Neo-2 (Neo2A-Neo2B pair[3]) which minimally signals unless reconstituted, repositioned the switch relative to split Neo-2 to reduce strain in state $\beta\gamma_c H_X$, then rigidly fused Neo2B to the switch C-terminus and connected Neo2A to the switch N-terminus with a flexible linker (Extended Data Fig. 8a). For several of these variants (especially ASNeo2_cp03), dissociation of γ_c from the active signaling complex dramatically accelerates under lower effector concentrations than required for the original design, suggesting that the effector-binding cleft is more intact (Extended Data Fig. 8d). γ_c dissociation from ASNeo2_cp08 is accelerated by 5700-fold, the highest fold change observed for any design here (Extended Data Fig. 8d).

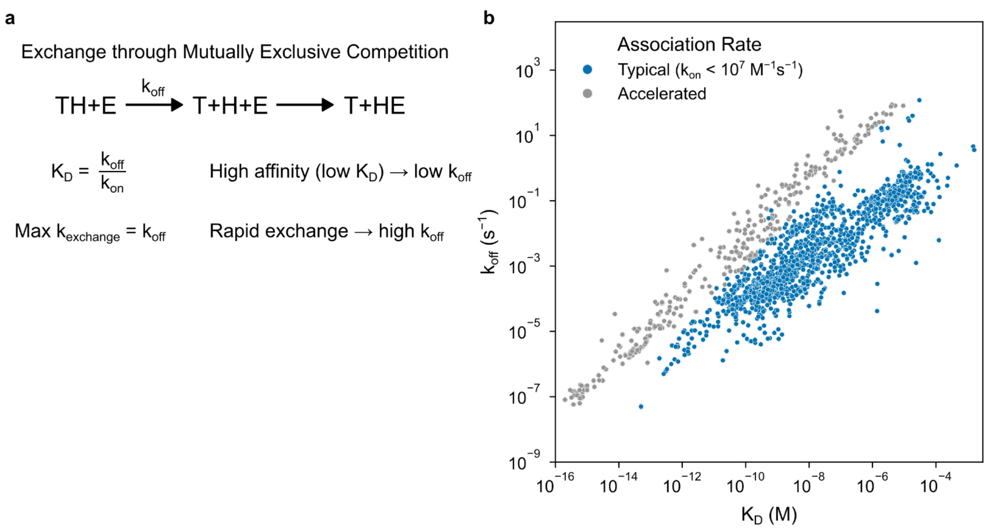

Supplementary Figure 1 | Relationship between affinity and exchange rate. a, For protein-protein interactions with typical diffusion-limited association rates in the 10^5–10^6 $M^{-1}s^{-1}$ range, high affinity (K_D < 1 nM) requires low off-rates ($k_{off} < 10^{-3}$ s^{-1}). This precludes rapid exchange through mutually exclusive competition, which requires high off-rates. **b**, Off-rate constant vs affinity for a set of natural and mutant proteins with typical on-rates (blue) and high on-rates (gray) depicting this tradeoff. Data obtained from the SKEMPI database[4].

High affinity and rapid exchange could be simultaneously achieved in binary interactions with high on-rates ($k_{on} > 10^7$ $M^{-1}s^{-1}$)[5], but these are usually caused by long-range electrostatic attraction and are not a general feature of protein-protein interactions. Although electrostatically accelerated association has been engineered into proteins[6], this is not possible in general with native targets because it requires patches of complementary surface charge on both binding partners.

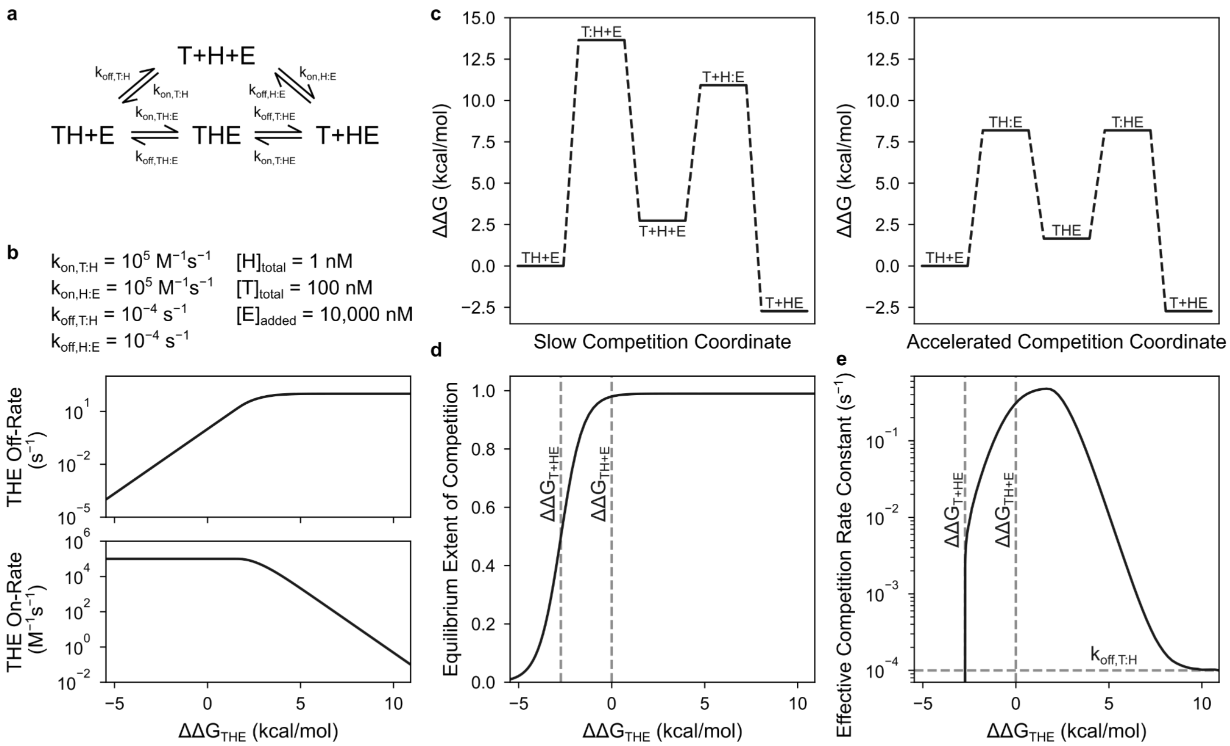

Supplementary Figure 2 | Dependence of facilitated dissociation on the energy of the ternary intermediate. **a**, Kinetic model of a facilitated dissociation system. We modeled the dynamics of a facilitated dissociation process for a range of ternary intermediate energies, simulated in this model by pre-equilibrating H and T then adding excess E. **b**, Model parameters used to simulate facilitated dissociation. We fixed the binary interaction rate constants, and we varied the ternary interaction rate constants to set the ternary intermediate energy. The plots show the relationships we chose between the ternary intermediate energy and the ternary interaction rate constants. These hold with physical intuition: low to moderate increases in ternary intermediate energy primarily affect dissociation rates, whereas high increases in ternary intermediate energy additionally affect association rates. **c**, Energy diagrams of slow mutually exclusive competition (left) and facilitated dissociation (right). Colons denote binding transition states between components. Binding E reduces the energy of the entire system, including the T dissociation transition state. Frustration between T and E causes the ternary intermediate energy to reduce less, lowering the barrier for T dissociation. **d**, Dependence of the equilibrium extent of the competition reaction on the ternary intermediate energy. If the ternary intermediate energy is too low (too little strain), the ternary complex becomes the dominant equilibrium state and T dissociation is not favored. **e**, Dependence of the competition rate constant on the ternary intermediate energy. The effective rate is defined as ln(2)÷{time to 50% extent of reaction} (this is undefined when $\Delta\Delta G_{THE} < \Delta\Delta G_{T+HE}$ because the final extent of reaction is less than 50%). The rate of competition peaks at an optimal ternary intermediate energy, then recedes to the basal rate of mutually exclusive competition as the ternary intermediate becomes increasingly energetically inaccessible.

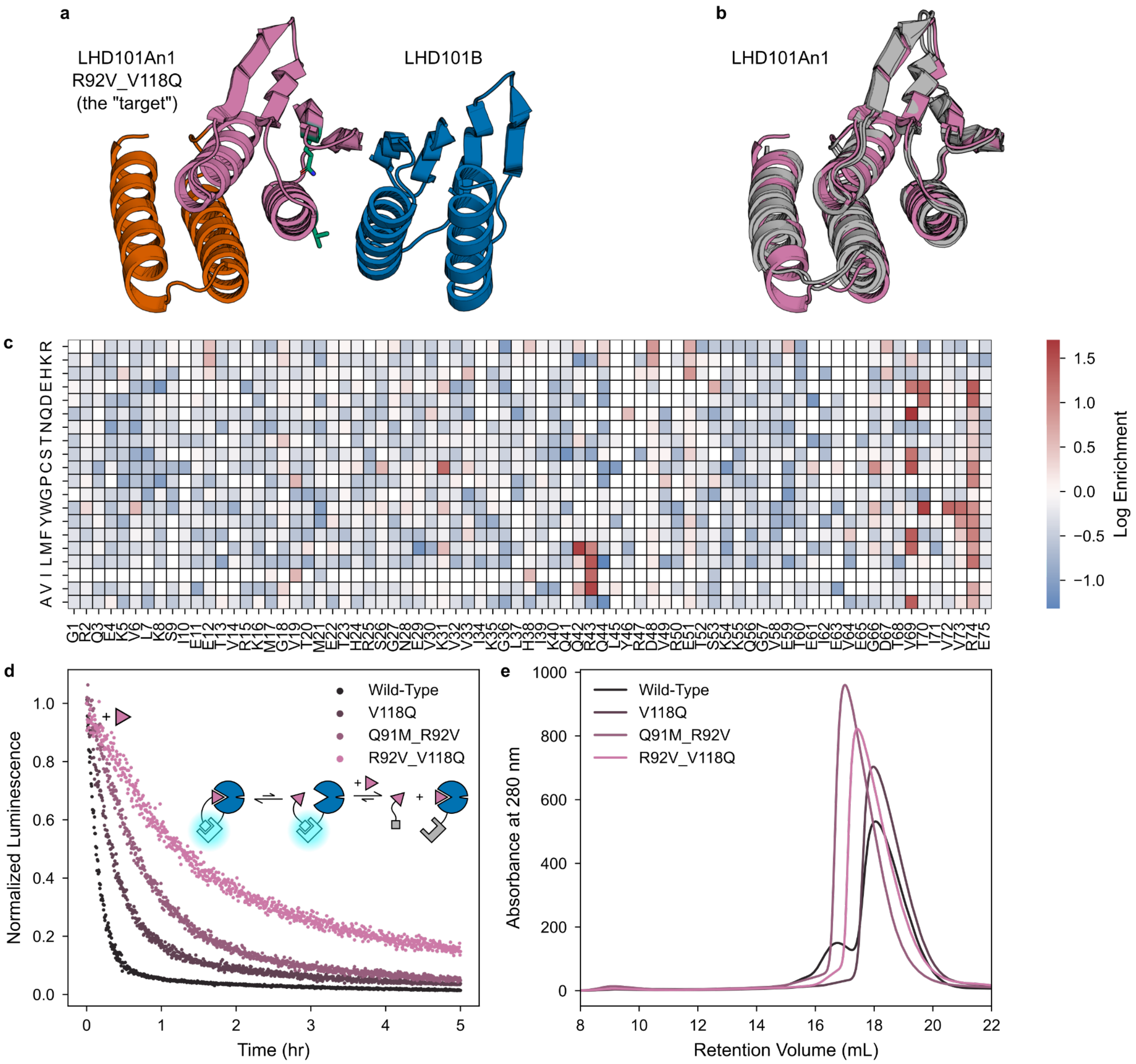

Supplementary Figure 3 | Design and characterization of the target. **a**, Design models of the target (pink/red) and binder (blue) in complex. Starting from LHD101A (pink), we built additional structure (red) to increase opportunities for steric clashing, resulting in the target protein "LHD101An1." Unless otherwise stated, "target" refers to LHD101An1 with affinity-enhancing mutations R92V and V118Q (green). **b**, Crystal structures of LHD101An1 (gray) overlaid with the design model (pink). **c**, Site-saturation mutagenesis heatmap showing the enrichment over wild-type of each LHD101A point mutation displayed on yeast sorted against 10 nM LHD101B. Mutations Q42M, R43V, and V69Q on LHD101A correspond to Q91M, R92V, and V118Q on LHD101An1. **d**, Dissociation time courses of preincubated 20 nM LHD101An1-SmBiT mutants and 100 pM AS0-LgBiT after adding 20 µM LHD101An1 competitor, showing the double mutant R92V V118Q especially reduces the intrinsic target-host off-rate. **e**, SEC purification runs of LHD101An1 mutants, performed on a Superdex 200 Increase 10/300 GL column with injection concentrations greater than 500 µM, showing these mutants remain well-behaved in solution.

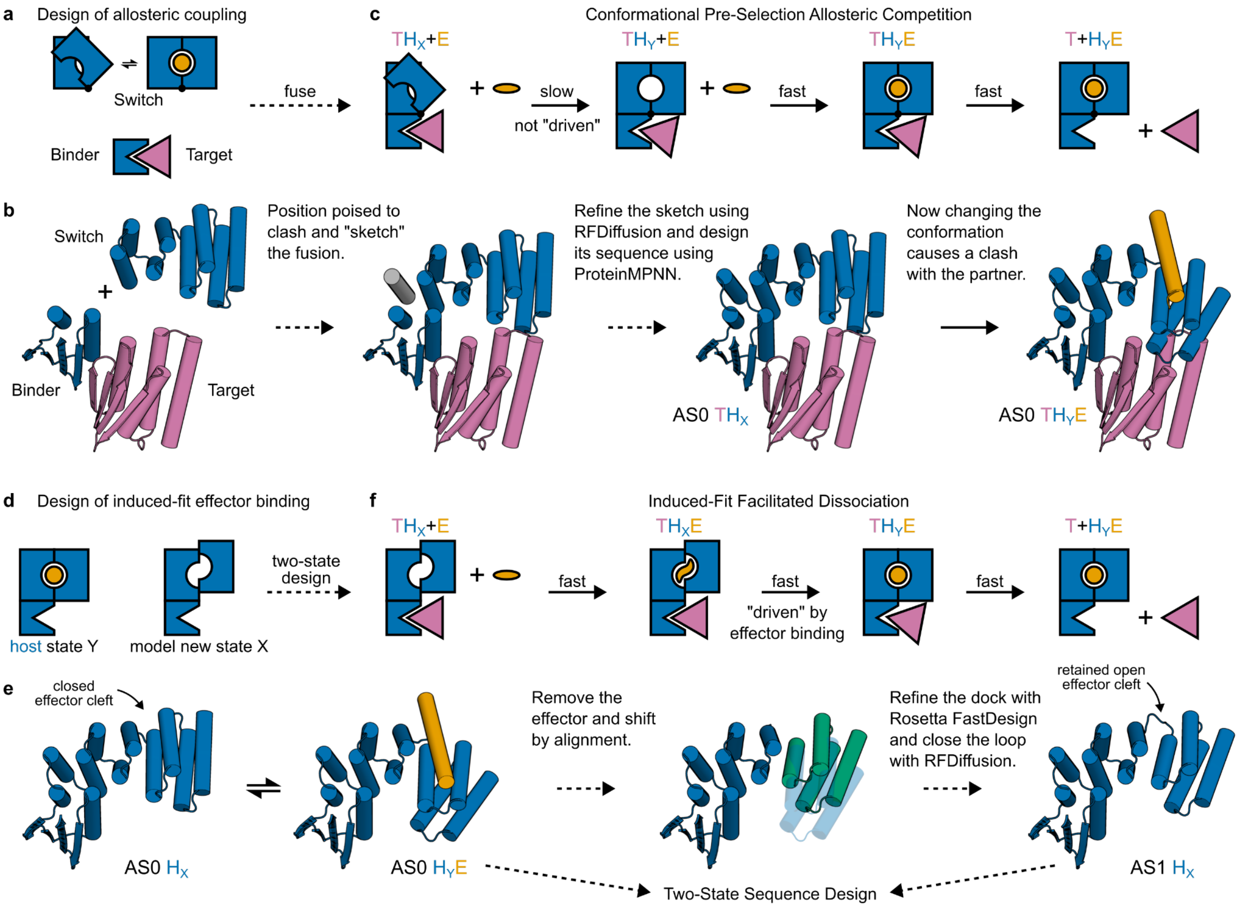

Supplementary Figure 4 | Approach to designing facilitated dissociation systems. a, **b**, Approach to designing host proteins by fusing a target binder and an effector-responsive switch. **a**, Cartoons of the starting components (a previously designed hinge switch and binder-target pair). **b**, Structural models showing how the switch and binder were fused to construct the first generation of hosts (including AS0). **c**, Cartoons showing the expected mechanism of competition, rate-limited by hinge opening. **d**, **e**, Approach to designing register-shift host proteins which retain an open cleft in state X. **d**, Cartoons of the register-shift hosts (AS0 state Y and a new state X with an open cleft). **e**, Structural models showing how the new state X was modeled from the state Y of AS0 to construct the second generation of hosts (including AS1). **f**, Cartoons showing the expected mechanism of competition, with a conformational change driven by effector binding.

The closed state X of the original hinge blocks effector binding, so the hinge must change conformation before the effector can bind: a conformational selection mechanism. In the absence of pre-existing conformational fluctuations of the target, spontaneous hinge opening against the target will generally be slow. The open state X of the new switches could allow weak effector binding which triggers the conformational change: an induced-fit mechanism. Our two-state design process generates sequences for the switch considering the state X alone and the effector-bound state Y. Thus, the switch is only designed to make strong interactions with the effector when in state Y, causing effector binding to promote the register shift to state Y.

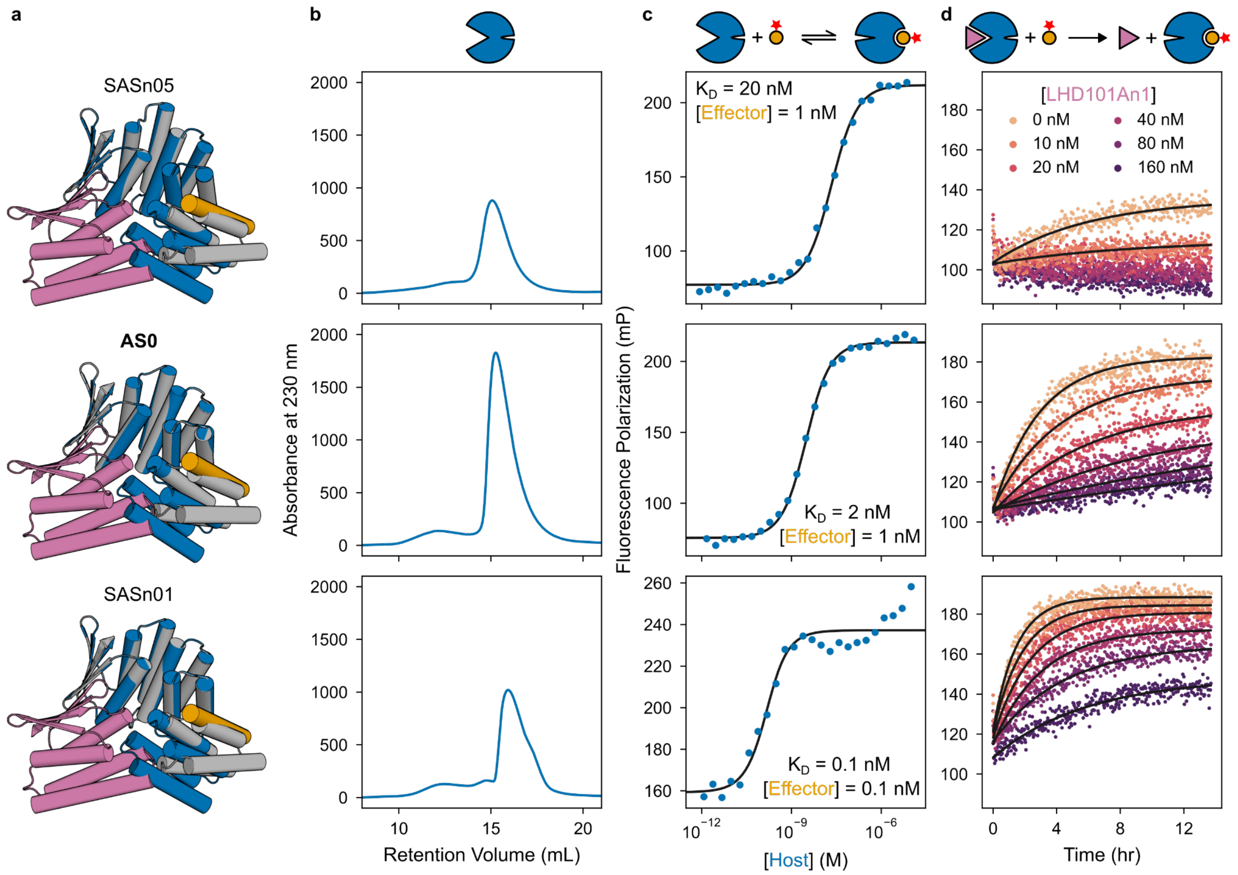

Supplementary Figure 5 | Negative allosteric coupling between target and effector. a, Design models of selected hinge-based host-effector complexes in state Y (blue and orange) aligned to the target (pink) and to the host in state X (gray). **c**, SEC host purifications performed on a Superdex 200 Increase 10/300 GL column. **d**, Fluorescence polarization (FP) titrations with a constant concentration of TAMRA-labeled effector and varying host concentrations. Data (blue) fit with a standard binding isotherm (black). **e**, Association of 20 nM TAMRA-labeled effector to 20 nM host against varying concentrations of the target LHD101An1, showing competition between target and effector binding. FP data (colors) fit with single exponentials (black).

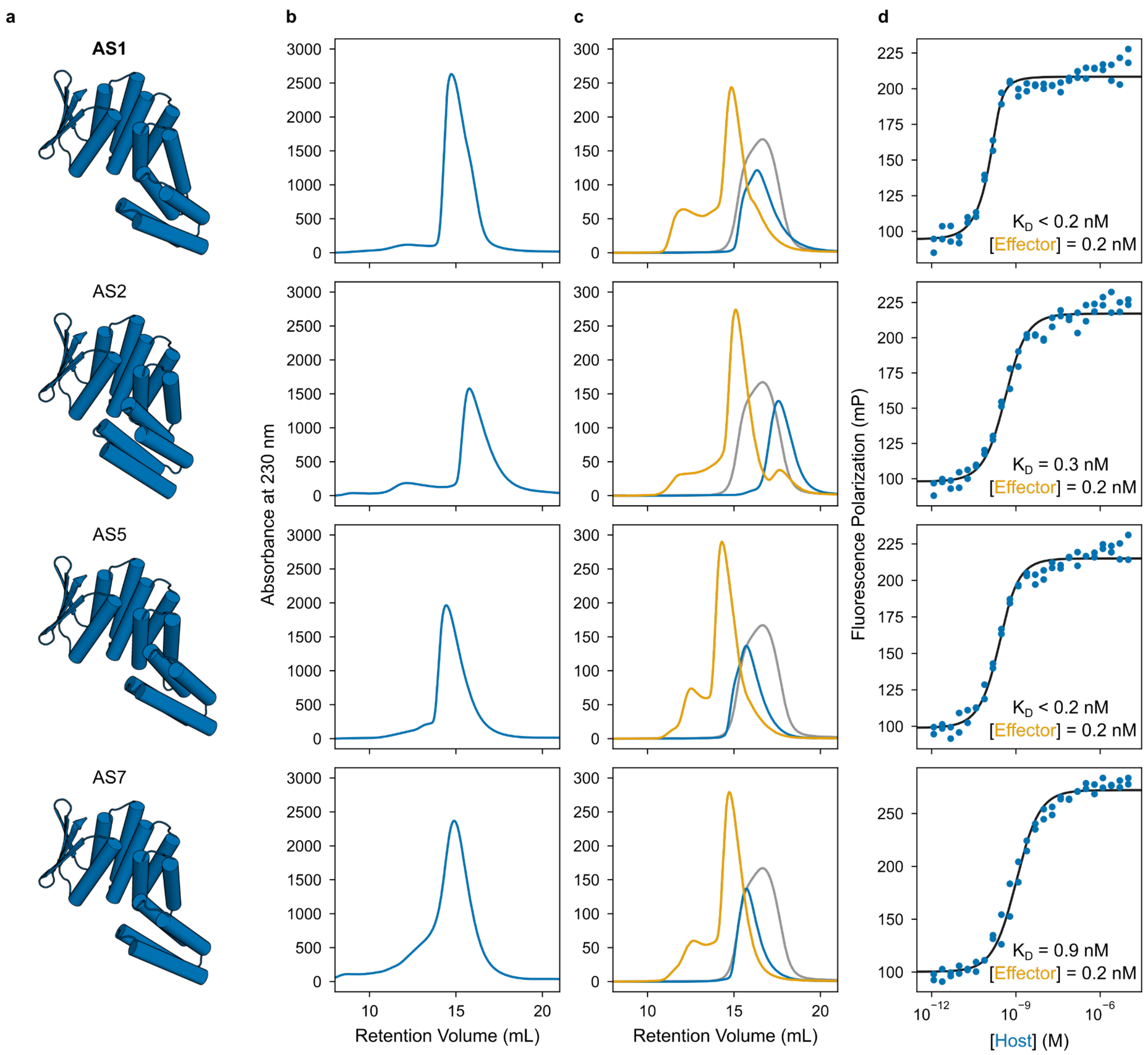

Supplementary Figure 6 | Initial characterization of register-shift host designs. a, Design models of selected register-shift host proteins in state X, showing the diversity of the new state X. **b**, SEC host purifications performed on a Superdex 200 Increase 10/300 GL column. **c**, SEC binding experiments performed on a Superdex 200 Increase 10/300 GL column. All components were injected at 20 μM. The mixtures of host and sfGFP-tagged effector (orange) run larger than the hosts alone (blue) or sfGFP-effector alone (gray), indicating host-effector binding. **d**, FP titrations with a constant concentration of TAMRA-labeled effector and varying host concentrations. Data (blue) fit with a standard binding isotherm (black).

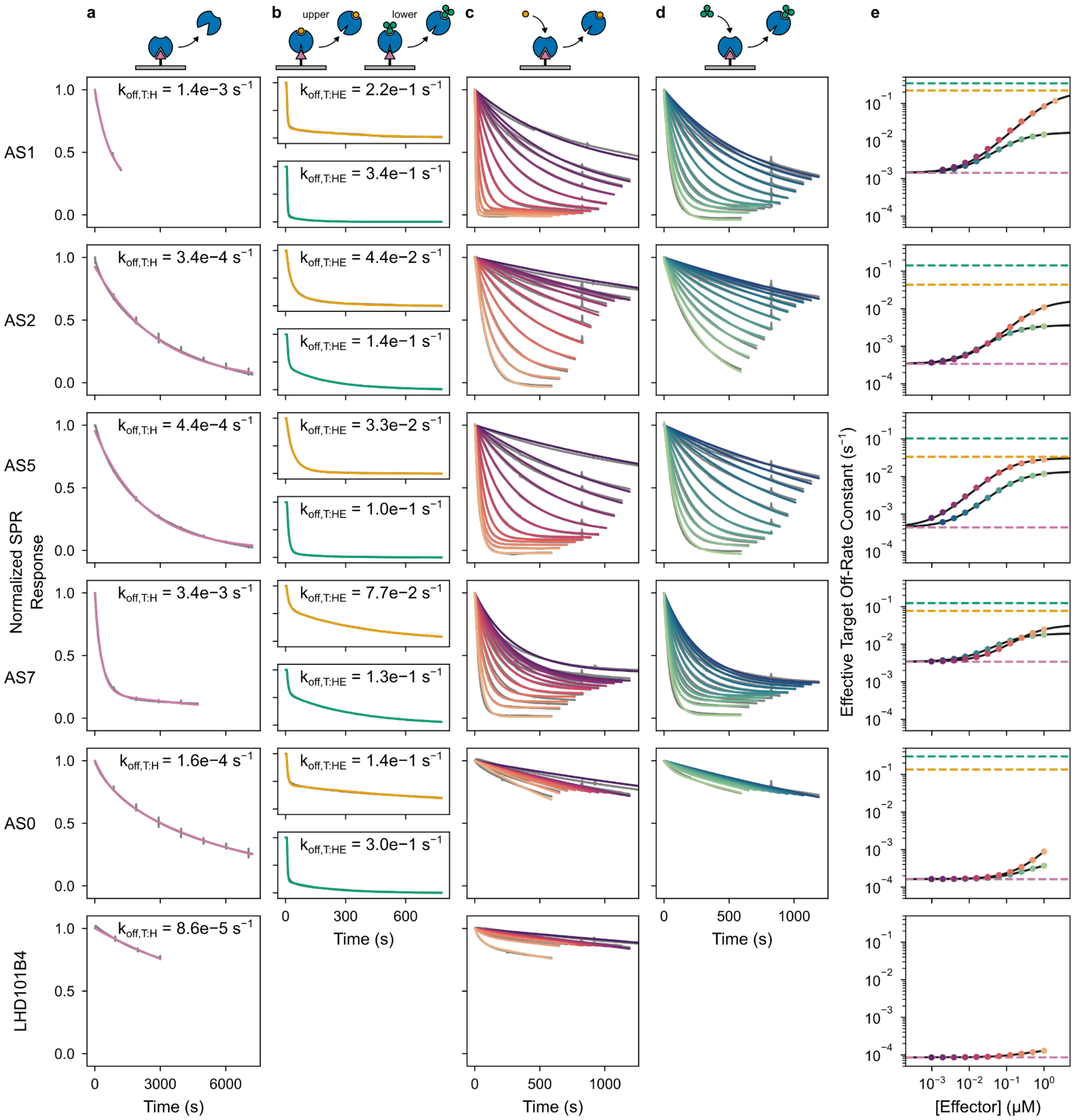

Supplementary Figure 7 | SPR characterization of facilitated dissociation in host designs. a, Slow dissociation of the target from the host in the absence of effector. Data (gray) fit with exponential decay functions (pink). For AS2 and AS5, single exponentials were used, whereas for the other designs, double exponentials were used to account for populations of host protein with different dissociation kinetics. For the double exponential fits, the reported dissociation rate constant, $k_{off,T:H}$, is the rate constant from the higher amplitude exponential in the fit. **b**, Faster dissociation of the target from the ternary complex with effector. In each row, the top plot corresponds to the peptide effector and the bottom plot to the 3hb effector. Data (gray) fit with double exponential decay functions (orange for peptide, green for 3hb) to account for a population

of target-host complex lacking the effector. The reported dissociation rate constant, $k_{off,T:HE}$, is the higher of the two rate constants in the fit. **c** and **d**, Effector concentration–dependent dissociation of the target upon addition of peptide (c) or 3hb (d) effector. Data (gray) fit (colors) as described in methods. **e**, Effective target off-rate constants computed from the model fit by ln(2)÷{half-time of target-host interaction} plotted against effector concentration (circles) and fit with hyperbolic equations (black lines). The orange circles correspond to the data with peptide effector from (c) and the green circles to the data with 3hb effector from (d). The pink line plots $k_{off,T:H}$ measured in (a), the orange line plots $k_{off,T:HE}$ with peptide effector measured in (b), and the green line plots $k_{off,T:HE}$ with 3hb effector measured in (b). With the peptide effector, the effective rate of the full facilitated dissociation pathway approaches the rate of target dissociation from the ternary intermediate, whereas with the 3hb effector, the effective rate of the full facilitated dissociation pathway approaches a lower value. As observed for AS1 (Fig. 2), this likely corresponds to peptide binding through induced-fit and 3hb binding rate-limited by a slower conformational selection. Once the effector is bound, however, the more deformable peptide less effectively strains the target interface (the target off-rate from the ternary complex is generally higher with the rigid 3hb than with the peptide) (Supplementary Table 2). At the top of **a**–**d**, cartoons show the arrangement of proteins relative to the SPR chip (gray). Only the experiments of panels (a) and (c) were performed for the LHD101B4 control. Also note that the data in gray is often hidden behind the colored fit curves.

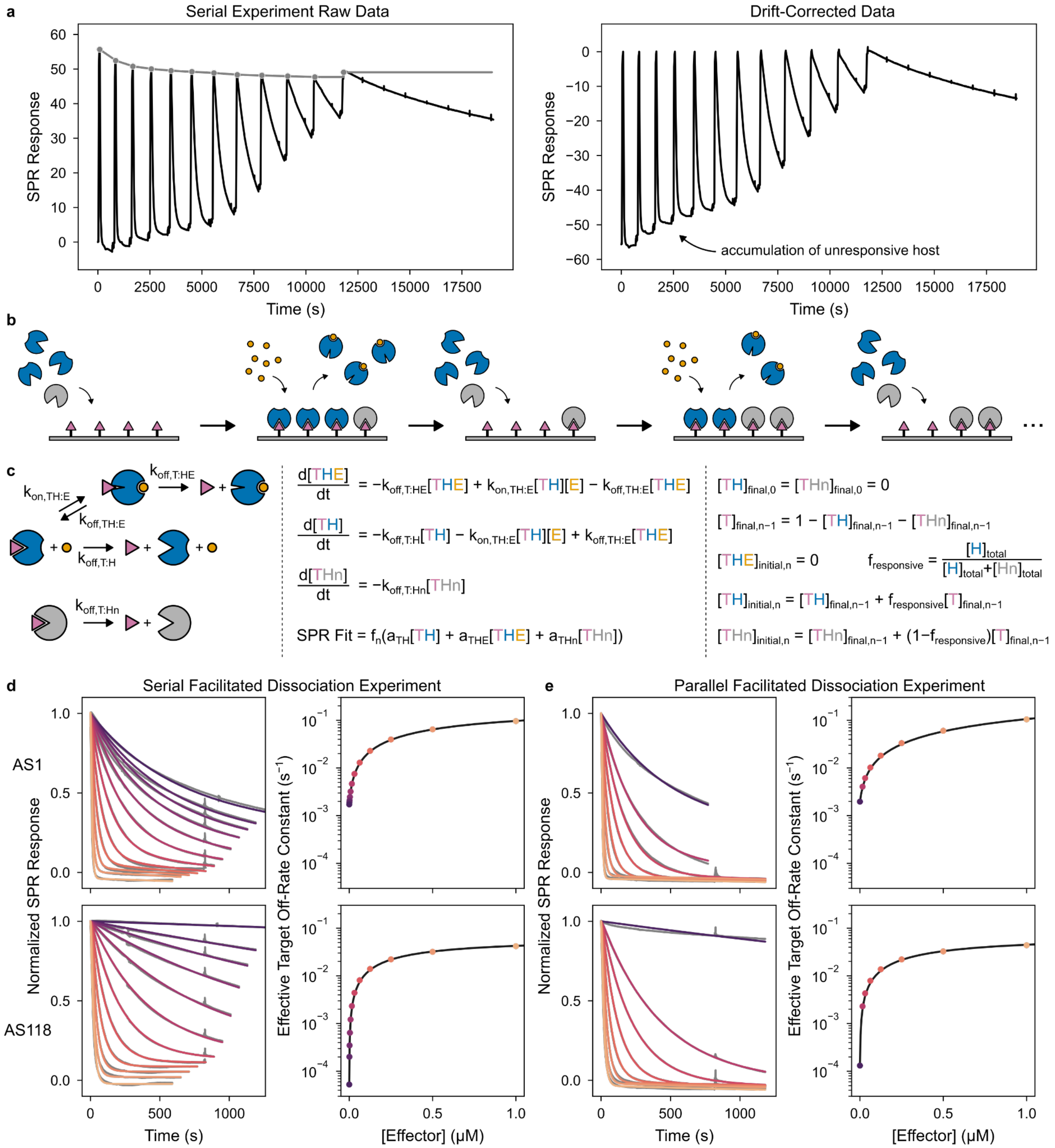

Supplementary Figure 8 | Fitting SPR facilitated dissociation data. a, (Left) raw SPR data (black) of a serial facilitated dissociation experiment involving multiple cycles of associating host then dissociating it with varying concentrations of effector. (Gray) approximate baseline drift estimated by interpolating between the peaks of host association (the SPR response should be the same when the surface is saturated with host). (Right) SPR data corrected for baseline drift by subtracting the gray baseline approximation from the raw SPR data. After each cycle, the dissociation trace plateaus at increasingly higher SPR response values, likely corresponding to

accumulation of a small population of host that is unresponsive to the effector—this could be due to partial degradation or misfolding induced by the strain in the ternary complex. **b**, Cartoons showing how a small population of unresponsive host (gray, "Hn") will accumulate on the surface after multiple facilitated dissociation cycles. **c**, Kinetic model fit to the facilitated dissociation experiment (see methods). (Left) cartoons depicting the state transitions which would affect the SPR response. (Middle) The system of differential equations corresponding to the kinetic model on the left, which can be fitted to the dissociation curve of each cycle. The bottom equation relates the concentrations of each state on the SPR surface to an SPR response. (Right) initial values for fitting this model to the dissociation curve of cycle *n*. **d** and **e**, (Left) fit data from serial (d) or parallel (e) facilitated dissociation experiments on AS1 (top row) or AS118 (bottom row). Data shown in gray and fits in colors. In the parallel facilitated dissociation experiment, each effector concentration is tested on a fresh SPR surface, minimizing accumulation of unresponsive host. Not needing to account for this, the model used to fit parallel facilitated dissociation data can be simpler. (Right) Effective target off-rate constants computed from the model fit by ln(2)÷{half-time of target-host interaction} plotted against effector concentration (circles, colors correspond to the fit dissociation traces in the left plots) and fit with a hyperbolic equation (black line). The target dissociation kinetics obtained from fitting the serial and parallel facilitated dissociation experiments agree within 4-fold, indicating that the serial experiment provides fairly accurate measurements of the target dissociation kinetics despite requiring a more complex model to fit.

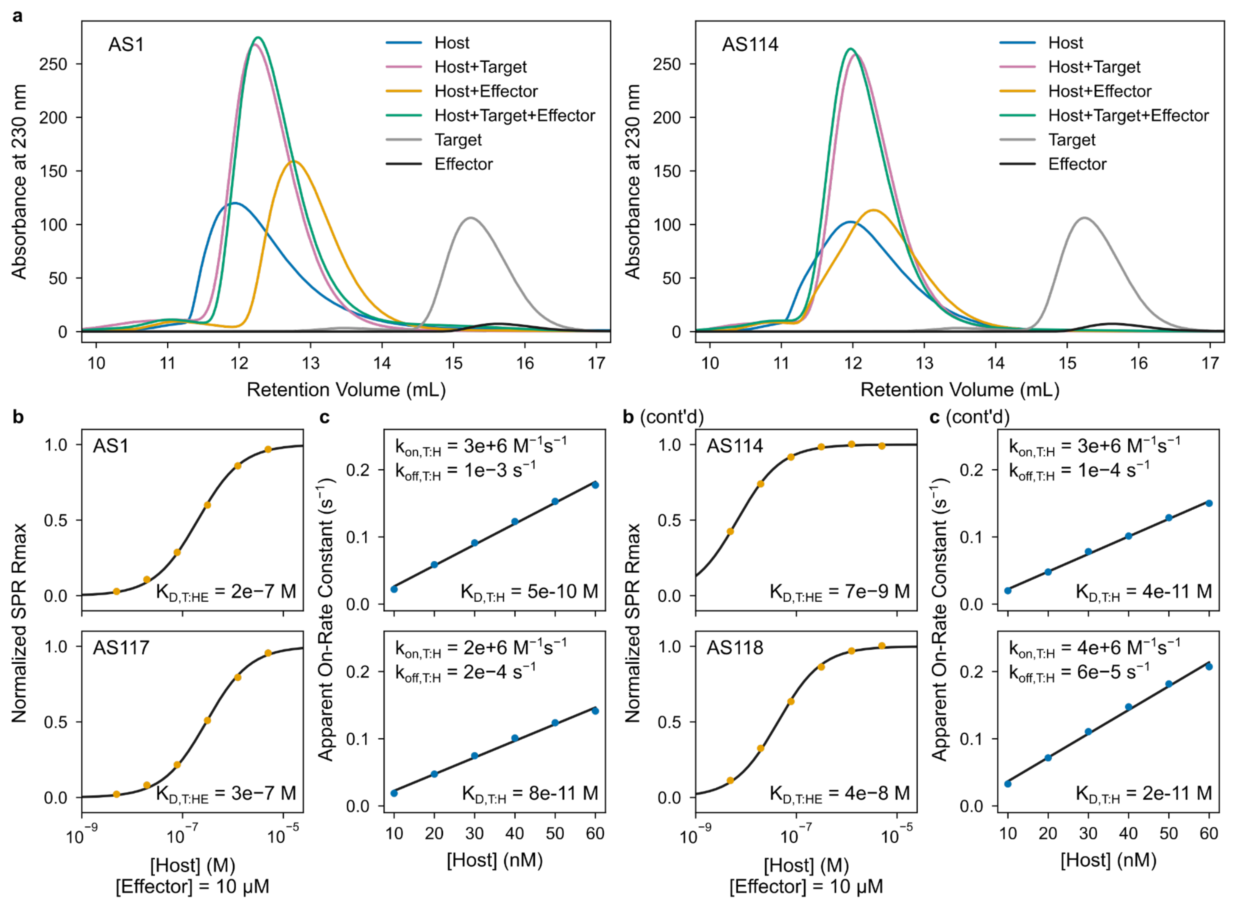

Supplementary Figure 9 | Stability of the AS1 and AS114 ternary complexes at high concentration. a, SEC binding experiments performed on a Superdex 75 Increase 10/300 GL column. All components were injected at 20 μM. In isolation, the target (gray) and effector (black) elute around 15–16 mL. When the host is included, depletion of signal in this range indicates target-host or host-effector binding. The hosts alone (blue) run large and are likely weak homodimers. Including either target (pink) or effector (orange) reduces homodimerization so the complex elutes later; signal past 15 mL is fully depleted, indicating complete binding to the host. Including both target and effector (green) near-fully (for AS1) or fully (for AS114) depletes signal past 15 mL, indicating both target and effector are bound to the host. For the AS1 ternary complex, the target was likely completely bound at the time of injection, but since the target dissociates from AS1 20-fold faster than from AS114 (Fig. 4a), some target dissociated while on the column, leaving the long tail extending past 15 mL. Thus, when both target and effector are present at sufficiently high concentrations, the ternary complex is the dominant state. **b** and **c**, Affinity measurements of the target binding the host-effector complex (b) and the host alone (c). **b**, Maximum response values with titrated host (preincubated with excess effector) plotted against host concentration. Data (points) fit with a standard binding isotherm. **c**, Apparent target:host on-rate constants vs. host concentration with a linear fit. The affinity is calculated as the ratio of the target:host off-rate constant (Fig. 2, Supplementary Fig. 11) and the on-rate constant from the fit.

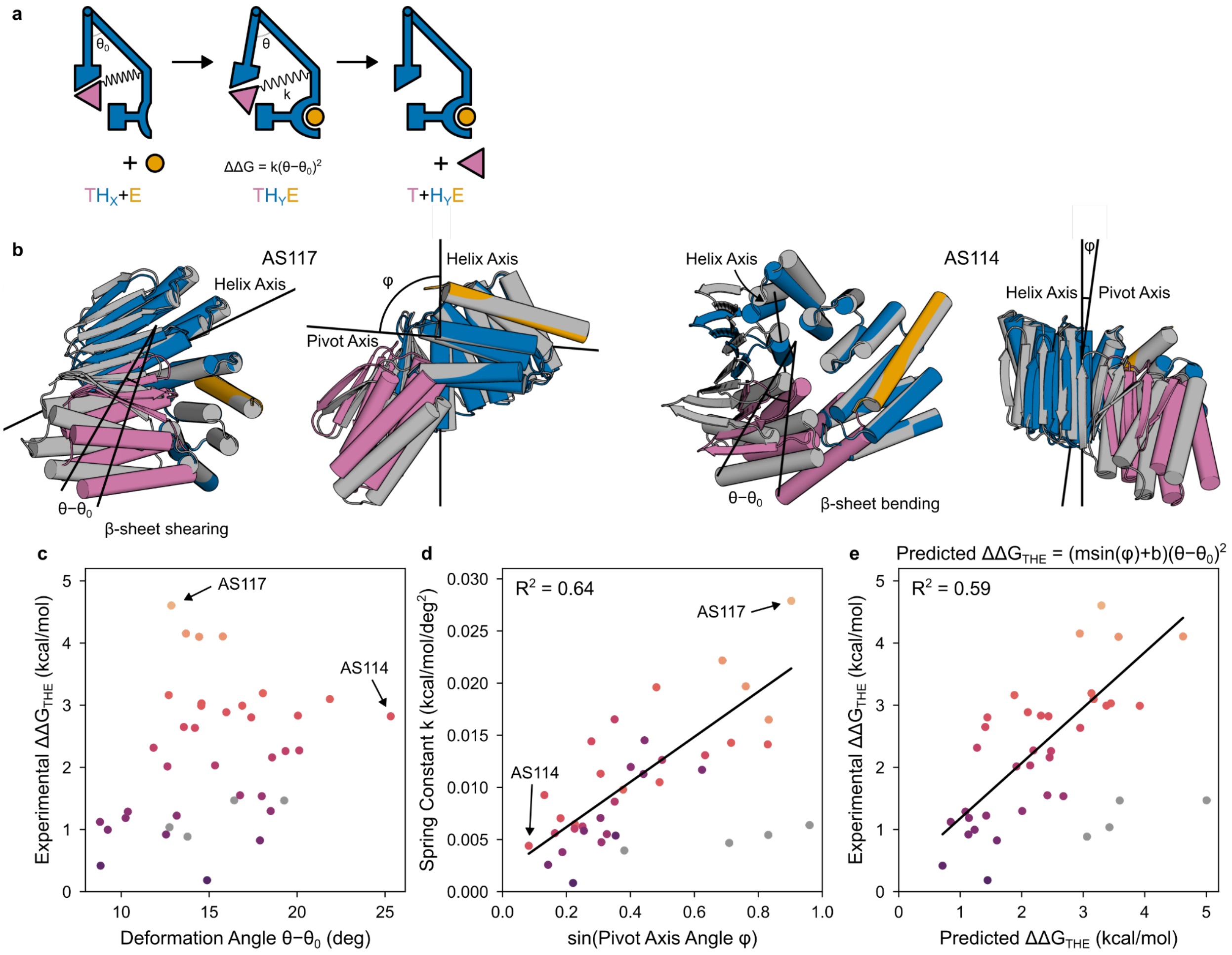

Supplementary Figure 10 | Modeling strain energy in the ternary complex. AF2 predicts AS1 and the fast variants like AS117 to globally deform in different directions from slower designs like AS114. Using a simple coarse-grained model based on the predicted magnitude and direction of the deformation relative to the secondary structure elements in the binder interface, across almost all designs, the predicted strain energy favoring partner dissociation correlates ($R^2 = 0.59$) with the strain energy estimated from the observed rate enhancement. Pearson's R^2 is calculated from linear least-squares regression.

a, Schematic of structural frustration resolved through strain in a facilitated dissociation pathway. Hooke's law can relate the energy of the ternary intermediate to mechanical properties of the protein.

b, Differences in ternary complex geometry for the fastest AS1 variant (AS117, left) and a slower variant which yet deforms more in the ternary complex (AS114, right). Design models of host·effector complex in state Y (blue and orange) aligned to the target (pink) showing the allosteric clash, and (gray) AF2 predictions of the ternary complex aligned to the switch showing how the clash resolves through global strain. To model the strain energy from these structures using Hooke's law, we measured the global deformation by the angle ($\theta-\theta_0$) the target pivots around

some axis (the "pivot axis") to move from its clashing position to its AF2-predicted strained position. The binder helix in the interface with the target tends to be positioned near the centerpoint of the deformation (the point around which the centroid of the target pivots), and the structure of this helix is the same for all variants, so we used the axis of this helix (the "helix axis") to approximate the orientation of the deforming secondary structure elements. For each variant shown, the left view places the pivot of the target within the plane of the page (so the pivot axis is normal to the page), and the right view places the helix axis and the pivot axis both within the plane of the page. In the left view, lines are drawn from the pivot axis through the centroids of the clashing and strained targets; the angle between these lines is the angle of global deformation ($\theta - \theta_0$). The right view shows the angle between the helix axis and the pivot axis (φ); this is large for the fast variant and small for the slow but highly deforming variant.

c, Lack of correlation between predicted magnitude of deformation and the experimental strain energy of the ternary complex for a set of host designs (AS1, AS2, AS5, AS7, and the AS1 variants, plotted as circles colored by their experimental strain energy except for AS101, AS115, AS119, and AS120 which were left out of the analysis in (c) and (d) and are colored gray—34 designs in total). The strain energy can be estimated from the observed accelerated off-rate constant as follows.

$$Experimental\ \Delta\Delta G_{THE} = RT\ ln\frac{K_{D,T:HE}}{K_{D,T:H,unstrained}} = RT\ ln\frac{k_{off,T:HE}k_{on,T:H,unstrained}}{k_{off,T:H,unstrained}k_{on,T:HE}}$$

Making the approximation that $k_{on,T:HE} = k_{on,T:H,unstrained}$ and does not vary with $\Delta\Delta G_{THE}$ (Supplementary Fig. 2) simplifies this expression.

$$Experimental\ \Delta\Delta G_{THE} = RT\ ln\frac{k_{off,T:HE}}{k_{off,T:H,unstrained}}$$

The accelerated off-rate constant $k_{off,T:HE}$ is assumed to be the maximum off-rate constant observed in the facilitated dissociation experiments (Supplementary Fig. 11). Since the main target-binder interface does not change across variants, the off-rate constant of the unstrained interface $k_{off,T:H,unstrained}$ should be a constant, k_{base}, with one exception: in some variants, the switch fusion may form additional stabilizing interactions with the target, reducing the base off-rate constant $k_{off,T:H}$ (also measured in the facilitated dissociation experiments). These interactions can likely still form in the strained ternary complex to reduce the accelerated off-rate constant $k_{off,T:HE}$ by the same factor. Thus, the smaller of k_{base} and $k_{off,T:H}$ is used for $k_{off,T:H,unstrained}$. For k_{base}, a value of 2e–4 s^{-1} was used because it gave the best correlation between predicted and experimental $\Delta\Delta G_{THE}$ described in panel (d). Notably, this value is quite close to the target off-rate constant from the unhindered binder fusion LHD101B4, 9e–5 s^{-1} (Supplementary Fig. 7).

d, Linear correlation between the "spring constant" k (which relates the experimental strain energy to the magnitude of the predicted deformation) and the perpendicularity of this deformation to the

secondary structure elements in the complex for this set of host designs (circles colored as in (b)). A linear regression on the colored points is plotted as a black line. The perpendicularity is computed as the sine of the angle between the pivot axis and the helix axis, and the spring constant is computed using Hooke's Law as follows.

$$Spring\ Constant\ k = \frac{\Delta\Delta G_{THE}}{(\theta - \theta_0)^2}$$

This relationship suggests that deforming in a stiff direction (against rather than around helices[2]) deforms the target interface in a more destabilizing direction or better localizes strain to the target interface instead of distributing the strain throughout the entire protein.

e, Agreement between the experimental strain energy and the strain energy estimated entirely from the predicted structure of the strained ternary complex using Hooke's Law for this set of host designs (circles colored as in (b)). A linear regression on the colored points is plotted as a black line. The spring constant was assumed to depend linearly on the deformation perpendicularity to the secondary structure elements, and the parameters of this correlation (m and b) were varied to fit the following expression to the experimental strain energies.

$$Predicted\ \Delta\Delta G_{THE} = (m\ sin(\varphi) + b)(\theta - \theta_0)^2$$

Sources of Error in this Analysis

First, in this simple energetic model of facilitated dissociation described here and in Supplementary Fig. 2, when the effector binds, the energy of the target dissociation transition state will decrease by the binding energy of the effector. When the effector and target are uncoupled, the energy of the ternary complex intermediate will decrease by the same amount so the activation barrier for target dissociation will not change. When strain between the target and effector is incorporated into the ternary intermediate, its energy will decrease less upon effector binding, reducing the activation barrier for target dissociation. This simple energetic model thus assumes that the activation barrier for target dissociation is directly related to the global energy of the ternary complex. In reality, acceleration of target dissociation will arise from local deformation at the target interface[7]. Thus, put differently, the simple energetic model assumes that the global deformation is smoothly distributed across the entire protein. This assumption allows us to directly relate the global deformation predicted by AF2 to the local deformation at the target interface measured by the accelerated target off-rate. In reality, the deformation is likely not smoothly distributed, and this may at least partially explain the imperfect correlation between the predicted and experimental strain energy of the ternary complex. This is exemplified by how the target dissociates more slowly from the AS1 ternary complex with the peptide effector than with the 3hb effector (Fig. 2e,f). Despite forming the same interactions and causing the same conformational change as the 3hb, the more deformable peptide appears to less effectively localize strain to the

target once in the ternary complex. Uneven distribution of strain in the ternary complex is also one possible mechanism underlying unidirectional competition in facilitated dissociation systems.

Second, our model estimating the protein stiffness anisotropy using the orientation of the secondary structure elements is likely oversimplified. Proteins have finer levels of structure than the orientation of their secondary structure elements. Much like a normal mode analysis, a more sophisticated model could estimate local spring constants for smaller regions of the protein, then compute a strain energy for each region from its predicted deformation.

Third, inaccuracies in the AF2 predictions of the strained ternary complex could cause much of the variation between the experimental and predicted strain energies. Clear cases of this, variants AS101, AS115, AS119, and AS120 (gray) which do not follow this correlation were left out of this analysis. We hypothesize that these variants can adopt an alternate, less-strained ternary complex conformation than the high-energy conformation predicted by AF2.

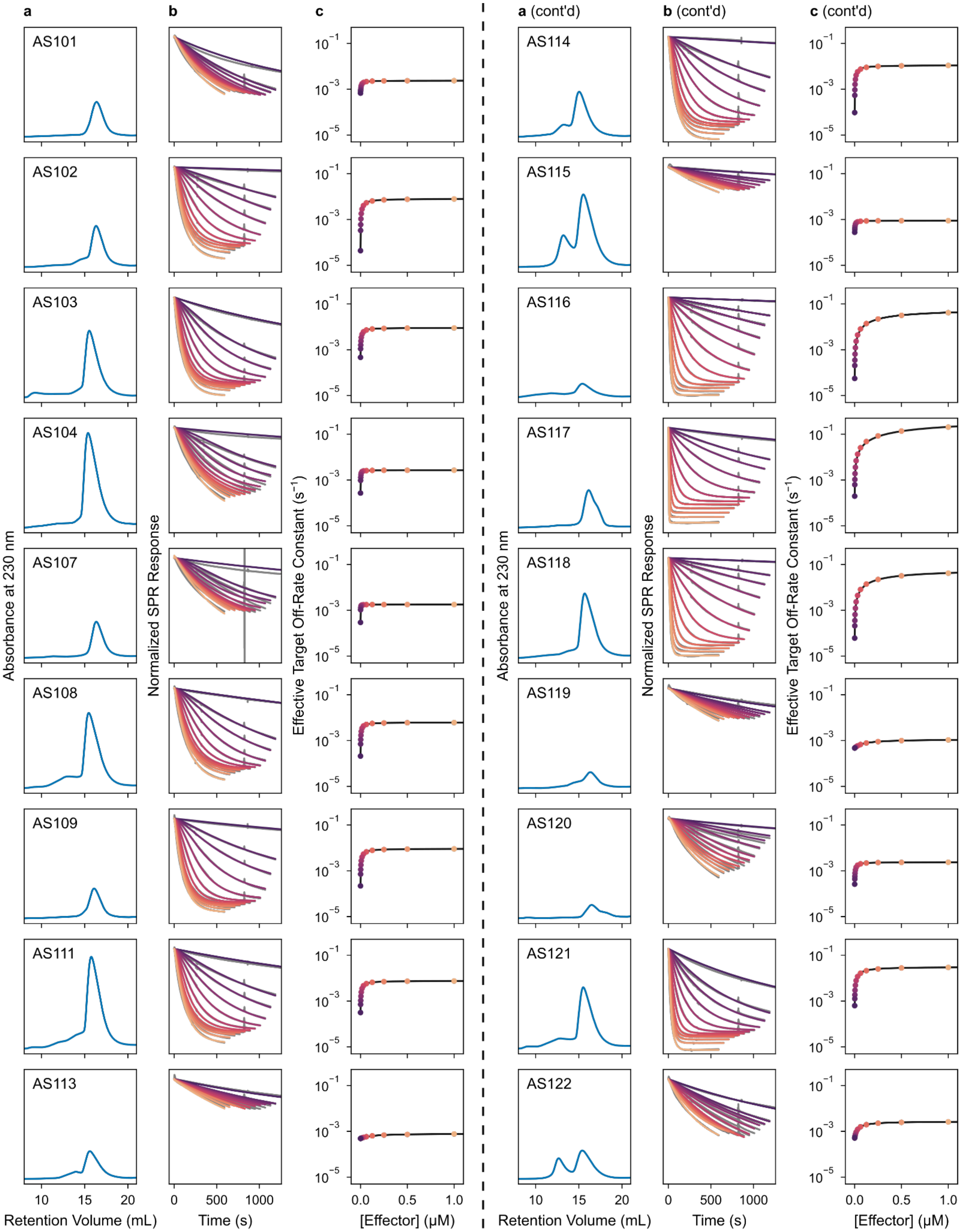
a
b
c
a (cont'd)
b (cont'd)
c (cont'd)
AS101
AS102
AS103
AS104
AS107
AS108
AS109
AS111
AS113
AS114
AS115
AS116
AS117
AS118
AS119
AS120
AS121
AS122
Absorbance at 230 nm
Normalized SPR Response
Effective Target Off-Rate Constant (s^{-1})
10^{-1}
10^{-3}
10^{-5}
10
15
20
0
500
1000
0.0
0.5
1.0
Retention Volume (mL)
Time (s)
[Effector] (μM)

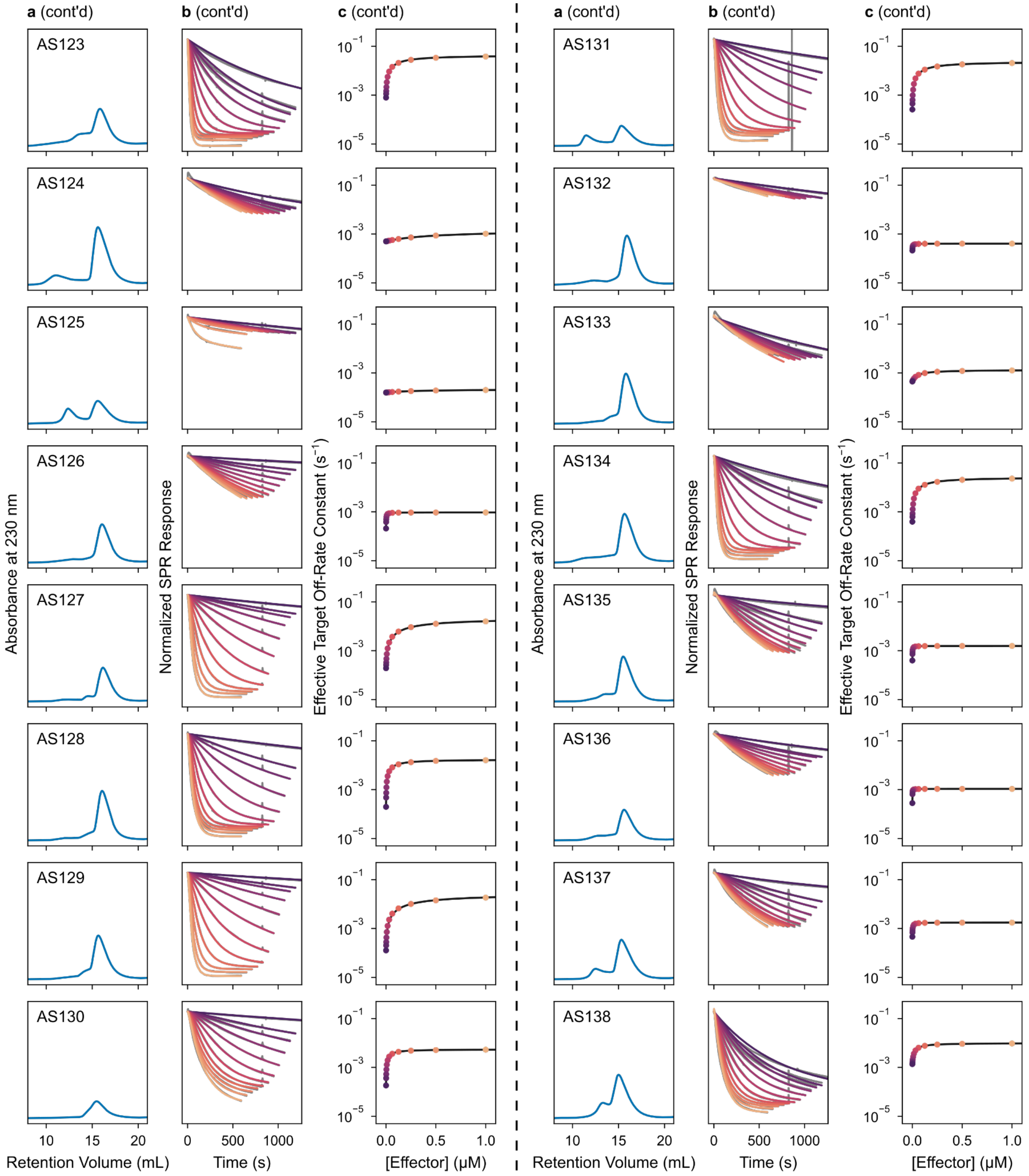

Supplementary Figure 11 | Characterization of AS1 variants. a, SEC host purifications performed on a Superdex 200 Increase 10/300 GL column. **b**, Effector concentration–dependent dissociation of the target upon addition of peptide effector. Data (gray) fit (colors) as described in methods. **c**, Effective target off-rate constants computed from the model fit by ln(2)÷{half-time of target-host interaction} plotted against effector concentration (circles) and fit with hyperbolic equations (black lines).

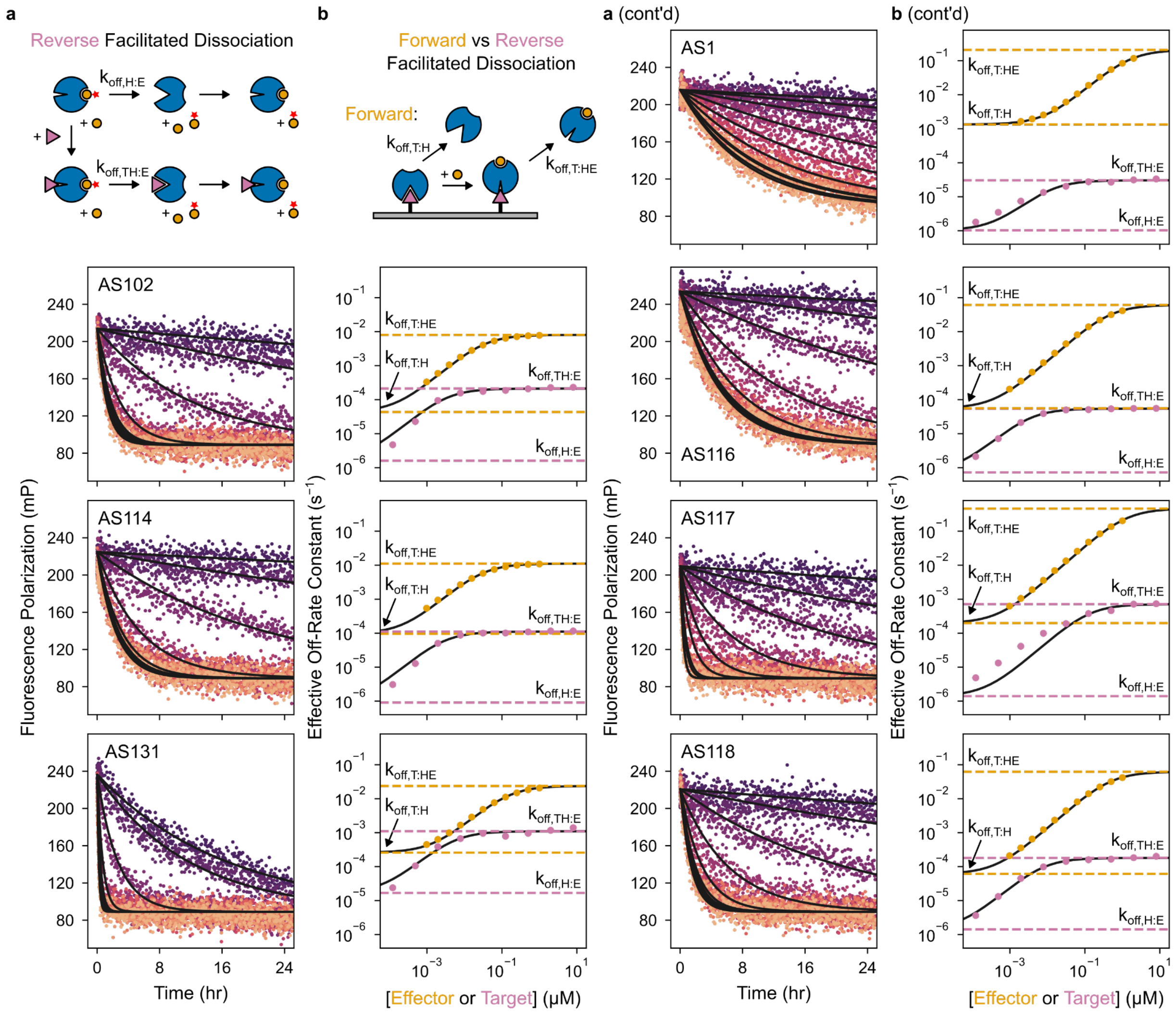

Supplementary Figure 12 | Reverse facilitated dissociation with selected AS1 variants. a, Target concentration–dependent dissociation of TAMRA-labeled effector, made irreversible by the presence of excess unlabeled effector. Fluorescence polarization data (colors) fit (black) with single exponentials. The cartoon shows the pathways of mutually exclusive competition and facilitated dissociation in the reverse competition assay. **b**, Comparison of effective facilitated dissociation rate constants in the forward (orange) and reverse (pink) directions. Forward facilitated dissociation data comes from Figs. 4 and Supplementary Fig. 11. The cartoon illustrates the SPR setup used to measure facilitated dissociation in the forward direction.

The left column shows designs with similar fold accelerations in the forward and reverse directions; the right column shows designs with significantly greater fold acceleration in the forward direction (see Extended Data Fig. 4).

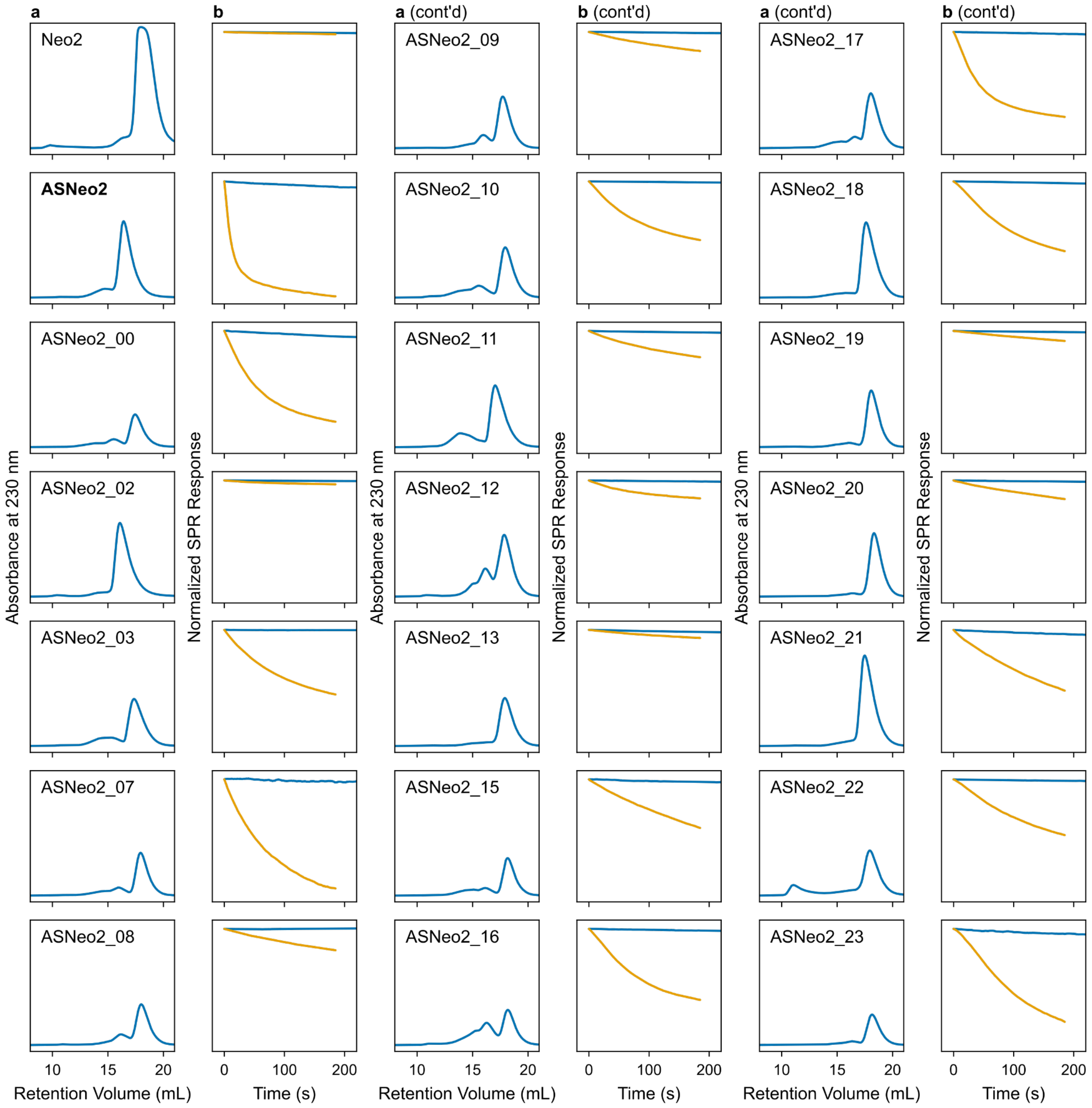

Supplementary Figure 13 | Characterization of initial switchable IL-2 mimic designs. a, SEC purifications performed on a Superdex 200 Increase 10/300 GL column. **b**, Slow dissociation of γ_c from the ASNeo2-IL-2R$\beta\gamma_c$ complex in the absence of effector (blue) and faster dissociation in the presence of effector (orange) as assessed by SPR.

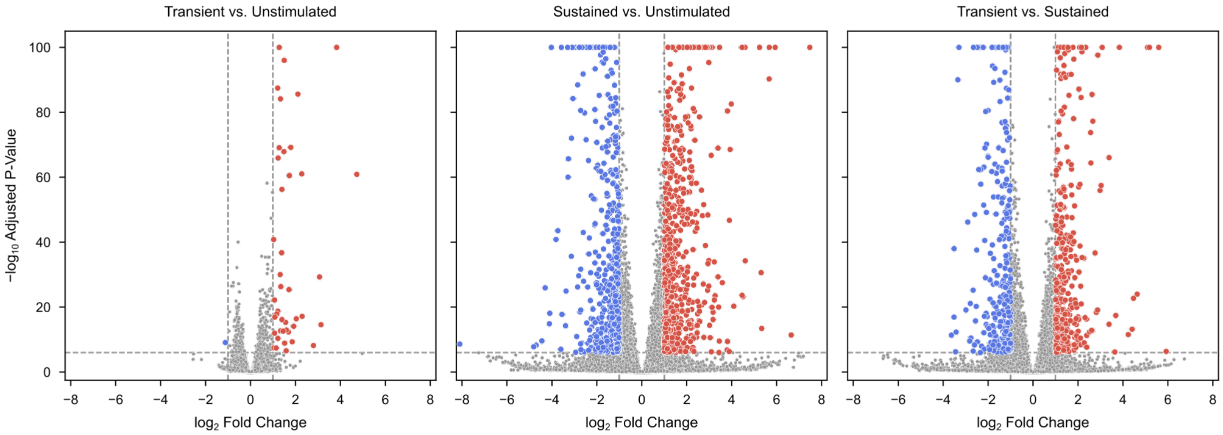

Supplementary Figure 14 | Differences in gene expression between no, transient, and sustained stimulation. Volcano plots showing differences in gene expression between different stimulation conditions. Red: significantly upregulated genes ($\log_2$FC > 1 and $P < 10^{-6}$), blue: significantly downregulated genes ($\log_2$FC < −1 and $P < 10^{-6}$), gray: genes not significantly differentially expressed ($|\log_2\text{FC}| < 1$ or $P > 10^{-6}$). $-\log_{10}$ Adjusted P-value was capped at 100.

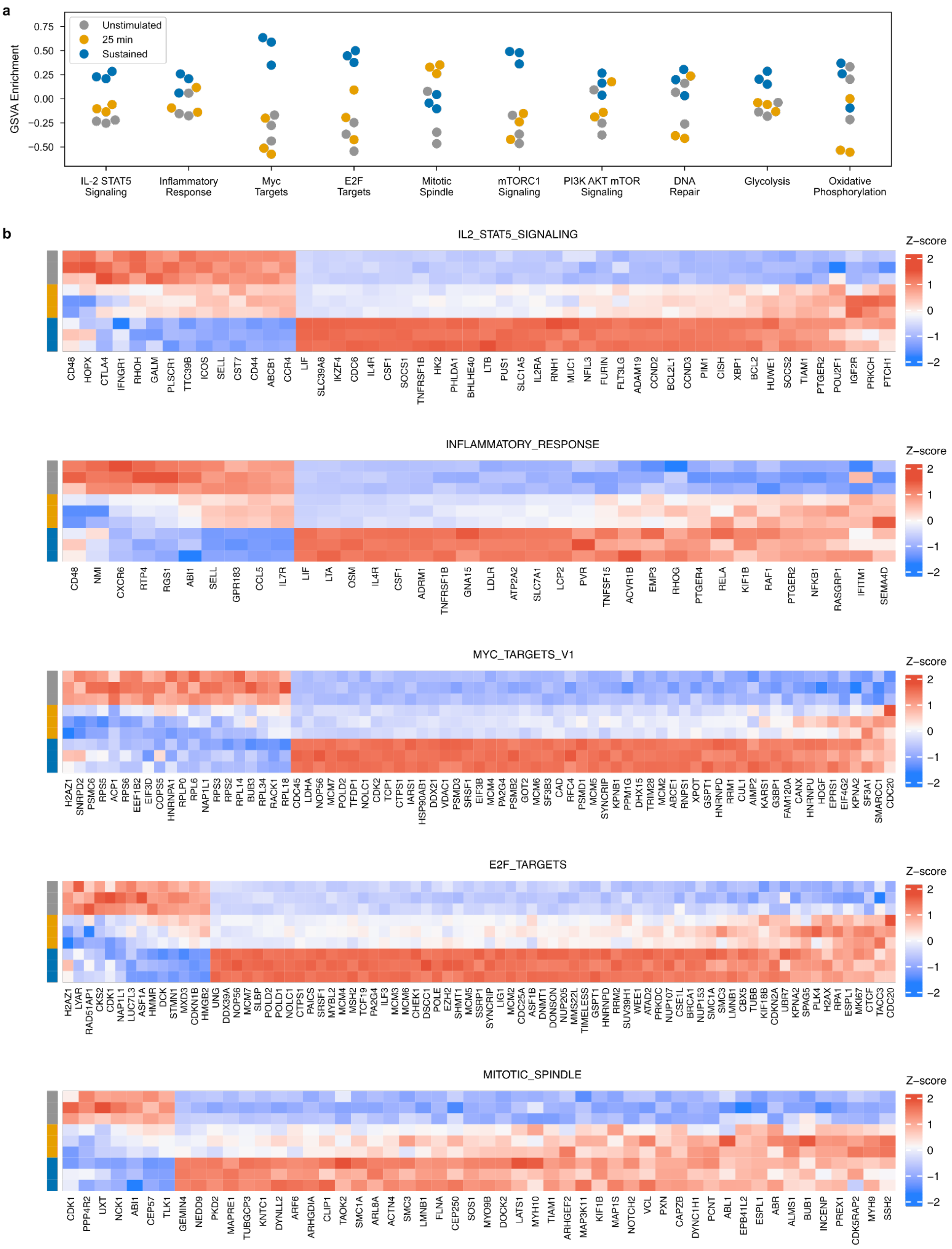
a
GSVA Enrichment
0.75
0.50
0.25
0.00
−0.25
−0.50
Unstimulated
25 min
Sustained
IL-2 STAT5 Signaling
Inflammatory Response
Myc Targets
E2F Targets
Mitotic Spindle
mTORC1 Signaling
PI3K AKT mTOR Signaling
DNA Repair
Glycolysis
Oxidative Phosphorylation
b
IL2_STAT5_SIGNALING
Z−score
2
1
0
−1
−2
CD48 HOPX CTLA4 IFNGR1 RHOH GALM PLSCR1 TTC39B ICOS SELL CST7 CD44 ABCB1 CCR4 LIF SLC39A8 IKZF4 CDC6 IL4R CSF1 SOCS1 TNFRSF1B HK2 PHLDA1 BHLHE40 LTB PUS1 SLC1A5 IL2RA RNH1 MUC1 NFIL3 FURIN FLT3LG ADAM19 CCND2 BCL2L1 CCND3 PIM1 CISH XBP1 BCL2 HUWE1 SOCS2 TIAM1 PTGER2 POU2F1 IGF2R PRKCH PTCH1
INFLAMMATORY_RESPONSE
Z−score
2
1
0
−1
−2
CD48 NMI CXCR6 RTP4 RGS1 ABI1 SELL GPR183 CCL5 IL7R LIF LTA OSM IL4R CSF1 ADRM1 TNFRSF1B GNA15 LDLR ATP2A2 SLC7A1 LCP2 PVR TNFSF15 ACVR1B EMP3 RHOG PTGER4 RELA KIF1B RAF1 PTGER2 NFKB1 RASGRP1 IFITM1 SEMA4D
MYC_TARGETS_V1
Z−score
2
1
0
−1
−2
H2AZ1 SNRPD2 PSMC6 RPS5 ACP1 RPS6 EEF1B2 EIF3D COPS5 HNRNPA1 RPLP0 RPL6 NAP1L1 RPS3 RPS2 RPL14 BUB3 RPL34 RACK1 RPL18 CDC45 LDHA NOP56 MCM7 POLD2 TFDP1 NOLC1 CDK2 TCP1 CTPS1 IARS1 HSP90AB1 DDX21 VDAC1 PSMD3 SRSF1 EIF3B MCM4 PA2G4 PSMB2 GOT2 MCM6 SF3B3 CAD RFC4 PSMD1 MCM5 SYNCRIP KPNB1 PPM1G DHX15 TRIM28 MCM2 ABCE1 RNPS1 XPOT GSPT1 HNRNPD RRM1 CUL1 AIMP2 KARS1 G3BP1 FAM120A CANX HNRNPU HDGF EPRS1 EIF4G2 KPNA2 SF3A1 SMARCC1 CDC20
E2F_TARGETS
Z−score
2
1
0
−1
−2
H2AZ1 LYAR RAD51AP1 CKS2 CDK1 NAP1L1 LUC7L3 ASF1A HMMR DCK STMN1 MXD3 CDKN1B HMGB2 UNG DDX39A NOP56 MCM7 SLBP POLD2 POLD1 NOLC1 CTPS1 PAICS SRSF1 MYBL2 MCM4 MSH2 TCF19 PA2G4 ILF3 MCM3 MCM6 CHEK1 DSCC1 POLE EZH2 SHMT1 MCM5 SSRP1 SYNCRIP LIG1 MCM2 CDC25A ASF1B DNMT1 DONSON NUP205 MMS22L TIMELESS GSPT1 HNRNPD RRM2 SUV39H1 WEE1 ATAD2 PRKDC NUP107 CSE1L BRCA1 NUP153 SMC1A SMC3 LMNB1 CBX5 TUBB KIF18B CDKN2A UBR7 KPNA2 SPAG5 PLK4 H2AX RPA1 ESPL1 MKI67 CTCF TACC3 CDC20
MITOTIC_SPINDLE
Z−score
2
1
0
−1
−2
CDK1 PPP4R2 UXT NCK1 ABI1 CEP57 TLK1 GEMIN4 NEDD9 PKD2 MAPRE1 TUBGCP3 KNTC1 DYNLL2 ARF6 ARHGDIA CLIP1 TAOK2 SMC1A ARL8A ACTN4 SMC3 LMNB1 FLNA CEP250 SOS1 MYO9B DOCK2 LATS1 MYH10 TIAM1 ARHGEF2 MAP3K11 KIF1B MAP1S NOTCH2 VCL PXN CAPZB DYNC1H1 PCNT ABL1 EPB41L2 ESPL1 ABR ALMS1 BUB1 INCENP PREX1 CDK5RAP2 MYH9 SSH2

b (cont'd)

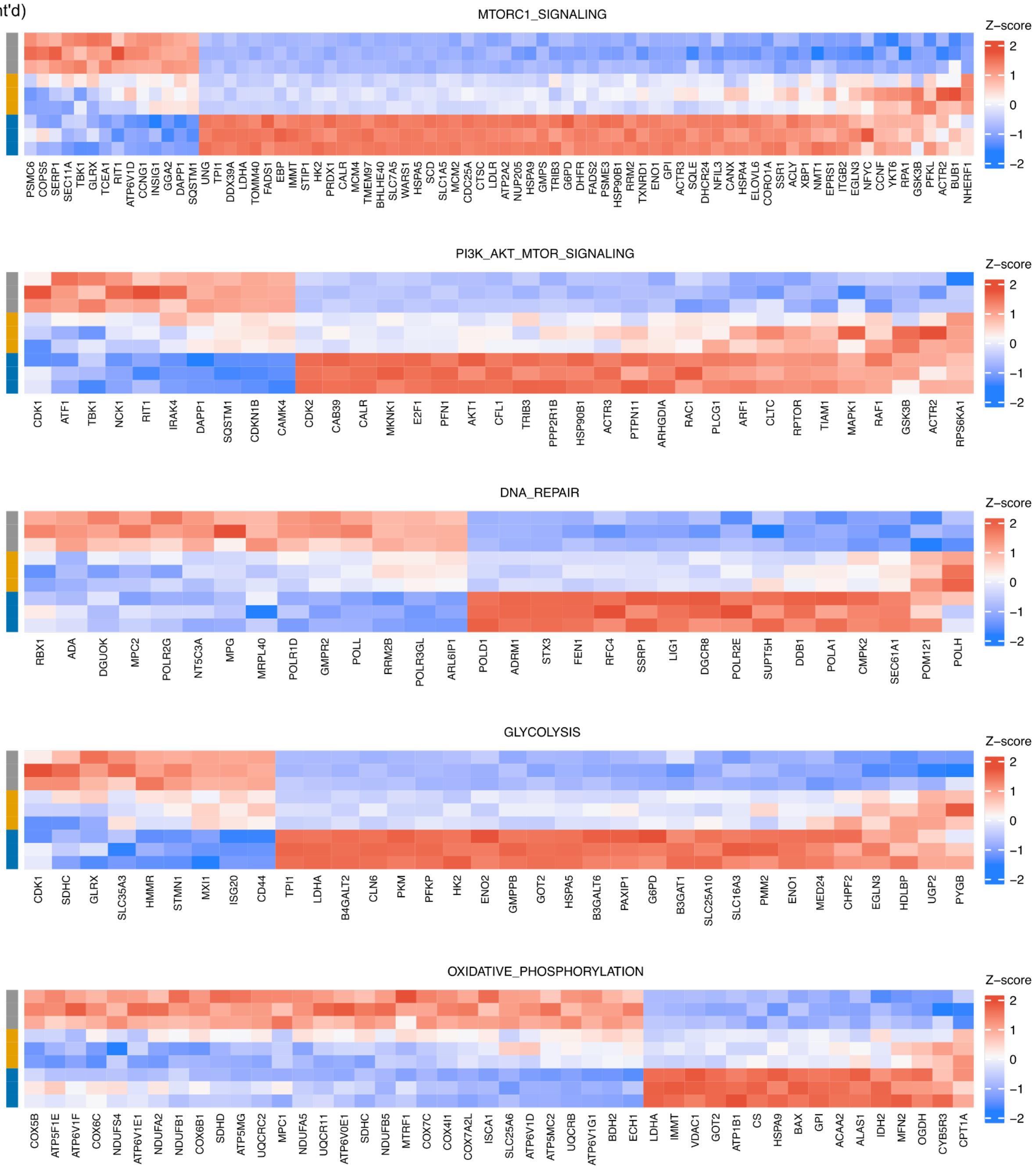

Supplementary Figure 15 | Variation in gene sets following different stimulation conditions. **a**, Gene set variation analysis (GSVA) for relevant hallmark genesets with statistically significant gene correlation, as identified by gene set co-regulation analysis (GESECA). Enrichment scores are shown for each replicate of each stimulation condition. **b**, Heatmaps of differentially expressed genes for each geneset.

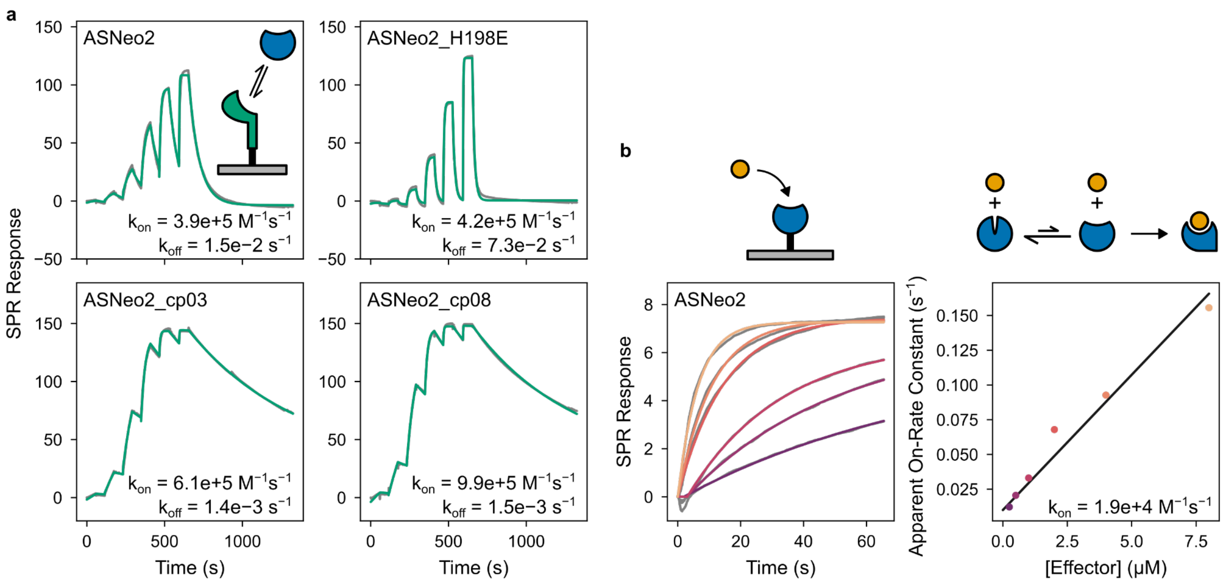

Supplementary Figure 16 | Additional kinetic characterization of ASNeo2 and variants. a, Single-cycle kinetics for ASNeo2 and selected variants binding IL-2Rβ. The mutant H198E reduces the ASNeo2:IL-2Rβ affinity by 5-fold. The cyclic permutations bind IL-2Rβ significantly stronger, perhaps because they lack helix 9 of the original ASNeo2 which may slightly sterically hinder binding to IL-2Rβ. **b**, (Left) effector association with ASNeo2; data (gray) fit with single exponentials (colors). (Right) apparent on-rate constants plotted against effector concentration (circles) and a linear fit. This on-rate is 10-fold slower than that between the effector and AS1, suggesting that the effector binding cleft is predominantly collapsed and the apparent on-rate is thus reduced by a rapid pre-equilibrium between the collapsed and intact conformations.

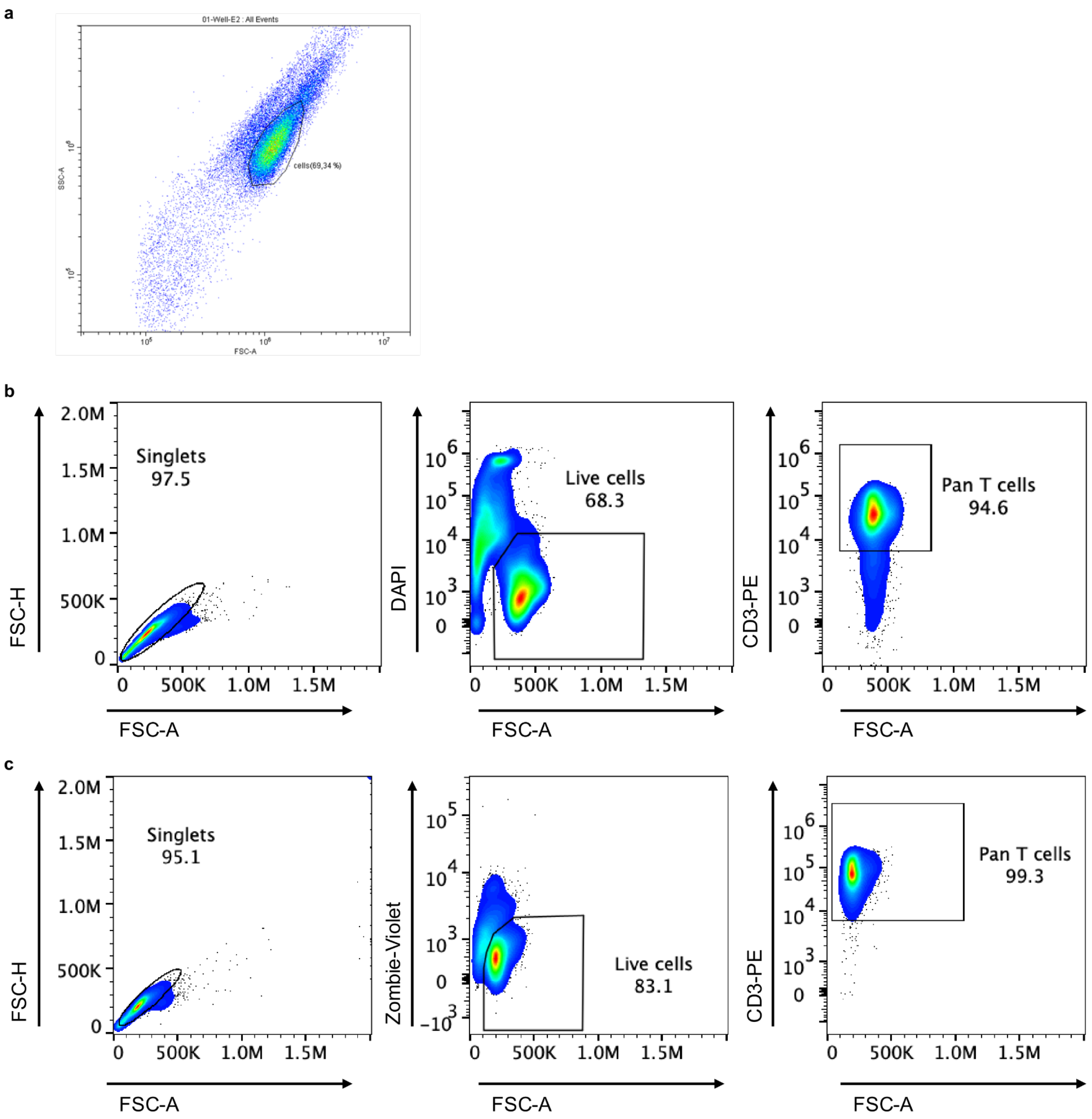

Supplementary Figure 17 | Flow cytometry gating. a, Gating strategy for YT cell identification in Fig. 5g,h based on forward/side scatter profiles. **b**, Gating strategy for T cell phenotyping shown in Fig. 5i,j and Extended Data Fig. 9a–d. Divided cells were gated within the $CD3^{+}$ T cell population after sequential gating on single, live cells. **c**, Gating strategy for T cell phenotyping in Extended Data Fig. 9e–g. Apoptotic and IL-2 downstream signaling markers were analyzed within $CD3^{+}$ T cells after gating on single, live cells.

Supplementary Table 1 | Kinetic parameters from forward and reverse facilitated dissociation experiments with target and peptide effector.

Design	$k_{off,T:H}$ (s^{-1})	$k_{off,T:HE}$ (s^{-1})	Peptide Effector EC_{50} (nM)	$k_{off,H:E}$ (s^{-1})	$k_{off,TH:E}$ (s^{-1})	Target EC_{50} (nM)
LHD101B4	8.6e−5	-	-	-	-	-
AS0	1.6e−4	1.4e−1	6500	-	-	-
AS1	1.4e−3	2.1e−1	130	1.0e−6	3.0e−5	2.2
AS2	3.4e−4	4.4e−2 (1.7e−2)	82	-	-	-
AS5	4.4e−4	3.1e−2	11	-	-	-
AS7	3.4e−3	7.7e−2 (3.3e−2)	130	-	-	-
AS101	6.7e−4	2.4e−3	5.1	-	-	-
AS102	4.3e−5	8.1e−3	2.2	1.6e−6	2.1e−4	0.26
AS103	4.7e−4	9.1e−3	3.5	-	-	-
AS104	2.7e−4	2.7e−3	0.84	-	-	-
AS107	2.9e−4	1.8e−3	0.38	-	-	-
AS108	2.1e−4	6.1e−3	2.4	-	-	-
AS109	2.2e−4	9.2e−3	3.4	-	-	-
AS111	3.2e−4	7.6e−3	4.1	-	-	-
AS113	4.9e−4	8.0e−4	110	-	-	-
AS114	9.6e−5	1.1e−2	2.7	9.0e−7	1.1e−4	0.23
AS115	2.8e−4	8.9e−4	3.3	-	-	-
AS116	5.6e−5	6.1e−2	14	7.3e−7	5.4e−5	0.37
AS117	2.0e−4	4.6e−1	25	1.4e−6	7.1e−4	5.5
AS118	6.1e−5	6.2e−2	14	1.4e−6	1.8e−4	0.63
AS119	4.7e−4	1.1e−3	94	-	-	-
AS120	2.6e−4	2.4e−3	4.2	-	-	-
AS121	6.3e−4	3.1e−2	7.9	-	-	-
AS122	5.1e−4	2.7e−3	29	-	-	-
AS123	7.9e−4	4.4e−2	19	-	-	-

AS124	5.0e−4	1.5e−3	490	-	-	-
AS125	1.6e−4	2.1e−4	320	-	-	-
AS126	2.1e−4	9.4e−4	1.5	-	-	-
AS127	1.9e−4	2.2e−2	33	-	-	-
AS128	2.0e−4	1.7e−2	7.5	-	-	-
AS129	1.3e−4	2.6e−2	28	-	-	-
AS130	1.8e−4	5.5e−3	5.9	-	-	-
AS131	2.6e−4	2.4e−2	15	1.7e−5	1.1e−3	0.62
AS132	2.1e−4	4.1e−4	2.4	-	-	-
AS133	4.5e−4	1.3e−3	46	-	-	-
AS134	3.9e−4	2.6e−2	16	-	-	-
AS135	4.0e−4	1.6e−3	1.3	-	-	-
AS136	2.8e−4	1.1e−3	0.57	-	-	-
AS137	4.6e−4	1.8e−3	2.5	-	-	-
AS138	1.4e−3	9.9e−3	16	-	-	-
ASNeo2	1.6e−4	2.4e−1	480	-	-	-
ASNeo2_cp03	1.5e−4	9.3e−2	36	-	-	-
ASNeo2_cp08	4.1e−5	2.4e−1	170	-	-	-
ASNeo2_cp12	6.4e−5	1.6e−1	280	-	-	-

Values in parentheses give the maximum rate constant of target dissociation induced by effector binding (the full facilitated dissociation pathway), if significantly different from the value of $k_{off,T:HE}$ measured directly by forming the ternary complex on the SPR chip. The EC_{50} is the concentration of effector required to achieve half the total acceleration of target dissociation on a log scale.

Supplementary Table 2 | Kinetic parameters from facilitated dissociation with 3hb effector.

**Design**	**$k_{off,T:H}$ (s^{-1})**	**k_{switch} (s^{-1})**	**3hb Effector EC_{50} (nM)**	**3hb Effector $k_{off,T:HE}$ (s^{-1})**	**Peptide Effector $k_{off,T:HE}$ (s^{-1})**
AS0	1.6e−4	5.1e−4	390	3.0e−1	1.4e−1
AS1	1.4e−3	1.7e−2	57	2.9e−1	2.1e−1
AS2	3.4e−4	3.7e−3	28	1.4e−1	4.4e−2
AS5	4.4e−4	1.4e−2	27	1.0e−1	3.3e−2
AS7	3.4e−3	1.9e−2	40	1.3e−1	7.7e−2
AS114	9.6e−5	-	-	4.7e−2	9.3e−3
AS118	6.1e−5	-	-	9.3e−2	7.4e−2

k_{switch} is the maximum rate constant of target dissociation induced by 3hb effector binding (the full facilitated dissociation pathway rate-limited by the switch conformational change). $k_{off,T:HE}$ is measured directly by forming the ternary complex on the SPR chip. Notably, $k_{off,T:HE}$ is nearly always higher with the 3hb effector than with the peptide, suggesting that the more rigid 3hb effector more effectively can localize strain to the target interface. The EC_{50} is the concentration of effector required to achieve half the total acceleration of target dissociation on a log scale.

Supplementary Table 3 | DEER experimental and fit parameters.

Construct	Sites	State	τ_2 (μs)	Δt (ns)	Scans	λ	SNR	t_0 offset (ns)	α
AS1	R35R1 E173R1	H	6.000	22	47	0.49	117	84.6	23.89
AS1	R35R1 E173R1	T+H	6.000	22	53	0.45	47	113.9	36.92
AS1	R35R1 E173R1	H+E	6.000	22	85	0.46	87	98.4	40.00
AS1	R35R1 E173R1	T+H+E	6.000	22	135	0.42	46	124.2	10.71
AS114	E31R1 E165R1	H	7.000	22	27	0.47	32	91.5	50.00
AS114	E31R1 E165R1	T+H	6.000	22	93	0.38	60	79.4	6.46
AS114	E31R1 E165R1	H+E	6.000	22	140	0.42	89	91.5	7.06
AS114	E31R1 E165R1	T+H+E	6.000	22	138	0.43	70	86.3	22.06

Δt - Pump pulse time step
λ - Modulation depth
SNR - Signal-to-noise
α - Smoothing parameter

Supplementary Table 4 | Sequences of designed proteins.

Design	Sequence
LHD101An1	EEAVRRRFEELLREALAFRERTGGRRETLEHAVRLARELAEFAASHPEFNRQEAVLLAIEL MVRAMGVTMETHRSGNEVKVVIKGLNIDEQRALYRAVRETSKIMGVETEIEVEGDTVTIVV RE
LHD101An1 _R92V	EEAVRRRFEELLREALAFRERTGGRRETLEHAVRLARELAEFAASHPEFNRQEAVLLAIEL MVRAMGVTMETHRSGNEVKVVIKGLNIDEQVALYRAVRETSKIMGVETEIEVEGDTVTIVV RE
LHD101An1 _V118Q	EEAVRRRFEELLREALAFRERTGGRRETLEHAVRLARELAEFAASHPEFNRQEAVLLAIEL MVRAMGVTMETHRSGNEVKVVIKGLNIDEQRALYRAVRETSKIMGVETEIEVEGDTQTIVV RE
Target: LHD101An1 _R92V _V118Q	EEAVRRRFEELLREALAFRERTGGRRETLEHAVRLARELAEFAASHPEFNRQEAVLLAIEL MVRAMGVTMETHRSGNEVKVVIKGLNIDEQVALYRAVRETSKIMGVETEIEVEGDTQTIVV RE
Effector	EERKKELAKEVIETAKKLIEKLAKEE
3hb Effector	SKEEAKKEFMELARKKAKEIEENPEKARELAEEALKELEKKYEELKKAGVPEKEALALYVI ALARVLIAKLAAEE
AS0	EMKEEIRRLAEELRERTKDEEVRELAREAARLAEESDDEEVKEVVKKALEAALKSKDEEVI RLLLLAAVLAAAAARSGSPEEKLEIAKKALELAMKSKDERVIRGALKAAVAAARSDDELALK TVKEALEKAMASKDERLIRAILAAAYAFALLAVAGASAERLKEAEAIVKELIAAAEKGASPQE LVLLVIEMMVKGMGVTMETHRSGNEVKVVIKGLHESQQEVLLEAVLFAAELMGVRVRIRF KGDTVTIVVRE
AS1	SMKEEIKRLAEELKEKTKNEEIKRLAEEAAELAERSDDPEVLEVVKKALEEALKSKNEEKIE LLLLVAVLVAEAGSVDAVEEKLEIALLALKLAEESKDPRIIRGALRAAIAALRSDDPLALKTVK EALERARASKDERLIRAILAAAYAFALLAVAGASAERLKEAEAIVKELIAAAEKGASPQELVL LVIEMMVKGMGVTMETHRSGNEVKVVIKGLHESQQEVLLEAVLFAAELMGVRVRIRFKGD TVTIVVRE
AS2	SMKEEIERLAEELARRSSDPEVRRLAREAARLARESDDPEVHEVVLRALRLALESSDEER VRLLLLAAVLVAEAARGGVPEEKLEIARLALELAREARDPRIKRGALRAALAALRSDDPLAL RTVREALERARASDDERLIRAILAAAYAFALLAVAGASAERLKEAEAIVKELIAAAEKGASPQ ELVLLVIEMMVKGMGVTMETHRSGNEVKVVIKGLHESQQEVLLEAVLFAAELMGVRVRIR FKGDTVTIVVRE
AS5	SLREEIRKLAEQLSEKYKDEEIRELAREAAELAEESDDPEVLELAYEALKKGLELEDEEKVK LILLAAVLAARVARGEVPEEKLEIALKALELAEASEDERIIRGALRAALAAARTDDPLALEVV LEALERAQASEDERLIRAILAAAYAFALLAVAGASAERLKEAEAIVKELIAAAEKGASPQELV LLVIEMMVKGMGVTMETHRSGNEVKVVIKGLHESQQEVLLEAVLFAAELMGVRVRIRFKG DTVTIVVRE
AS7	SMKEEIRKLAEELSKKTKDEEVRKLAREAAELAEKSEDPEVHKIVLEALKEALKSKDEAKIR LLLLAAVLAAKAISEGAVLEKLEIAKKALELAEESEDERIKRGALEAAYAALRSDDPLALKTV KEALERARESEDERLIRAILAAAYAFALLAVAGASAERLKEAEAIVKELIAAAEKGASPQELV LLVIEMMVKGMGVTMETHRSGNEVKVVIKGLHESQQEVLLEAVLFAAELMGVRVRIRFKG

	DTVTIVVRE
AS101	SMKEEIKRLAEELKEKTKNEEIKRLAEEAAELAERSDDPEVLEVVKKALEEALKSKNEEKIE LLLLVAVLVAEAGSVDAVEEKLEIALLALKLAEESKDPRIIRGALRAAIAALRSDDPEVLKAVK EALEKARASKDEKIIYVLLTLAEVAARFGWSYEQAKRIIDAFLELRKKASPQEVVLAVIERTV EAMGVTMETHRSGNEVKVVIKGLHESQQEALLRIVLATAEAEGVRVRIRFKGDTVTIVVRE
AS102	SMKEEIKRLAEELKEKTKNEEIKRLAEEAAELAERSDDPEVLEVVKKALEEALKSKNEEKIE LLLLVAVLVAEAGSVDAVEEKLEIALLALKLAEESKDPEIIRGALRAAIAALRSDDPGVLKVVK EALELARASKDEAIIKILLSLAYAIAEIKASEEEGKKALKEVLEAYKKLKPQEVVLKVIELTVKL MGVTMETHRSGNEVKVVIKGLHESQQEILLRVVLSTAEALGVRVRIRFKGDTVTIVVRE
AS103	SMKEEIKRLAEELKEKTKNEEIKRLAEEAAELAERSDDPEVLEVVKKALEEALKSKNEEKIE LLLLVAVLVAEAGSVDAVEEKLEIALLALKLAEESKDPRIIRGALRAAIAALRSDDPEVLKFVK EALEKARASKDEKKIIVILTLAYIAALFKFDEEQLKLLKEAMEILESKLPPQEKVLKVIELTVKA MGVTMETHRSGNEVKVVIKGLHESQQEALLEIVLSTAEYEGVRVRIRFKGDTVTIVVRE
AS104	SMKEEIKRLAEELKEKTKNEEIKRLAEEAAELAERSDDPEVLEVVKKALEEALKSKNEEKIE LLLLVAVLVAEAGSVDAVEEKLEIALLALKLAEESKDPRIIRGALRAAIAALRSDDPEVLKAVK YALELARASKDEKVILVLLTIAYEAALLGWPYEKVKKIIDKYLEAIKSGLSPQEKVLLVIEQTV EAMGVTMETHRSGNEVKVVIKGLHESQQEALLRIVLTTAEAEGVRVRIRFKGDTVTIVVRE
AS107	SMKEEIKRLAEELKEKTKNEEIKRLAEEAAELAERSDDPEVLEVVKKALEEALKSKNEEKIE LLLLVAVLVAEAGSVDAVEEKLEIALLALKLAEESKDPRIIRGALRAAIAALRSDDPVVLKLVK EALELARASKDERAIRFILVLAEAAARLKVDEELAKEAKELMERARAGELSQELVLKTIELLV KAMGVTMETHRSGNEVKVVIKGLHESQQELLLRVVLETAEAAGVRVRIRFKGDTVTIVVR E
AS108	SMKEEIKRLAEELKEKTKNEEIKRLAEEAAELAERSDDPEVLEVVKKALEEALKSKNEEKIE LLLLVAVLVAEAGSVDAVEEKLEIALLALKLAEESKDPRIIRGALRAAIAALRSDDPEVLKFVK EALELARASKDEKIIRLLLTLAYAAAKLNADEEEAKRFKEVMEKALAGGDPQEIVLAAIEALV AAMGVTMETHRSGNEVKVVIKGLHESQQEFLLEVVLETAEAMGVRVRIRFKGDTVTIVVR E
AS109	SMKEEIKRLAEELKEKTKNEEIKRLAEEAAELAERSDDPEVLEVVKKALEEALKSKNEEKIE LLLLVAVLVAEAGSVDAVEEKLEIALLALKLAEESKDPRIIRGALRAAIAALRSDDPEILKAVK EALELARASKDEKKILILLTTAEAGARLGKPELIENFIKRWEELEKEGASPQEKVLASIEVTV EAMGVTMETHRSGNEVKVVIKGLHESQQEALLRIVLTTAEAQGVRVRIRFKGDTVTIVVRE
AS111	SMKEEIKRLAEELKEKTKNEEIKRLAEEAAELAERSDDPEVLEVVKKALEEALKSKNEEKIE LLLLVAVLVAEAGSVDAVEEKLEIALLALKLAEESKDPEIIRGALRAAIAALRSDDPEVLKIVK EALELARASKDEKIIVHILELAYIAAKTGNAEEGKKAIEKIKKAYEEGVKPQEKVLLSIEETVKI MGVTMETHRSGNEVKVVIKGLHESQQELLLRYVLRTAELEGVRVRIRFKGDTVTIVVRE
AS113	SMKEEIKRLAEELKEKTKNEEIKRLAEEAAELAERSDDPEVLEVVKKALEEALKSKNEEKIE LLLLVAVLVAEAGSVDAVEEKLEIALLALKLAEESKDPEIIRGALRAAIAALRSDDPEVLKAVK YALELARASKDELLIRVALLLAEAAARLGLPAEKVKKALKEIEKAVKELPPQELVLKVIEATVE LMGVTMETHRSGNEVKVVIKGLHEEQQEALLRIVLTTAEETGVRVRIRFKGDTVTIVVRE
AS114	SMKEEIKRLAEELKEKTKNEEIKRLAEEAAELAERSDDPEVLEVVKKALEEALKSKNEEKIE LLLLVAVLVAEAGSVDAVEEKLEIALLALKLAEESKDPEIIRGALRAAIAALRSDDPEVLKAVK EALEEARASKDEAEIKTLLEAAELAARLGNVEIVKKVKEALKKVKEEKAPPQERVLAVIEAT VEAMGVTMETHRSGNEVKVVIKGLHEEQQEGLLRLVLTTAEAEGVRVRIRFKGDTVTIVV RE

AS115	SMKEEIKRLAEELKEKTKNEEIKRLAEEAAELAERSDDPEVLEVVKKALEEALKSKNEEKIELLLLVAVLVAEAGSVDAVEPKLEVALAALKLAEESKDPEVIRLALRLAIRALREGLSEEDLRVLAEIFKYLLESGLSEEEKAALLGIADLGWRLGLSAEEIKEIVKKLAEELKEKSPQEKVLLVIERAVELMGVTMETHRSGNEVKVVIKGLHESQQELLLRLVLEVAEASGVRVRIRFKGDTVTIVVRE
AS116	SMKEEIKRLAEELKEKTKNEEIKRLAEEAAELAERSDDPEVLEVVKKALEEALKSKNEEKIELLLLVAVLVAEAGSVDAVEEKLEIALIALKLAEESKDPKIIRAALRAAIRILRADPPEETLEKAVEILKKAYEEGADPEVIAVLLIAAAVAFELGLPEEKLEEYMEKVEKALKEGKRQEAVLLIIELLVDGMGVTMETHRSGNEVKVVIKGLHESQQEFLLETVLEVAEVLGVRVRIRFKGDTVTIVVRE
AS117	SMKEEIKRLAEELKEKTKNEEIKRLAEEAAELAERSDDPEVLEVVKKALEEALKSKNEEKIELLLLVAVLVAEAGSVDAVEPKLEAALIALKIAEESKDPRIIRAALRAAIRYLRQEVPEEVAKKVIEILKKAWEEGAPPEVLIILMLVAAVYFEGGKFEKAPEAVERALKLWKEGAPPQELVLLVIEAAVEAMGVTMETHRSGNEVKVVIKGLHESQQEWLLEVVLLAAEAAGVRVRIRFKGDTVTIVVRE
AS118	SMKEEIKRLAEELKEKTKNEEIKRLAEEAAELAERSDDPEVLEVVKKALEEALKSKNEEKIELLLLVAVLVAEAGSVDAVEPKLEIALIALKGAEESKDPKKIRAMLRAAIRVLRAPDTPAAEALLKRLVEAIKEKKLSLEQVVALALIVAALLEAGVPEEEALERLERALEKMEKASPQEIVLLVIEELVYAMGVTMETHRSGNEVKVVIKGLHESQQERLLEVVLLVAEATGVRVRIRFKGDTVTIVVRE
AS119	SMKEEIKRLAEELKEKTKNEEIKRLAEEAAELAERSDDPEVLEVVKKALEEALKSKNEEKIELLLLVAVLVAEAGSVDAVEPKLEIALLALKIAEESKDPELIRAALRAAIRALRTEDEEAARVVAELLKKLEEEGALSEKELVVLMLAAAKMMEAGVEPEELRKALEEMAKKLKGASEQEKVLLLIEAAVKAMGVTMETHRSGNEVKVVIKGLHESQQEELLRIVLEAAEVLGVRVRIRFKGDTVTIVVRE
AS120	SMKEEIKRLAEELKEKTKNEEIKRLAEEAAELAERSDDPEVLEVVKKALEEALKSKNEEKIELLLLVAVLVAEAGSVDAVEPKLEVALLALKIAEESKDPEKIRLALRSAIGILRLSTTPEREEVVKEMIKLIKEEKLSPEIAVALLSILEAALREGLPAEEAKKLLKELAEKLKGASPQERVLLVIEALVKVMGVTMETHRSGNEVKVVIKGLHESQQELLLRHVLGFAEAEGVRVRIRFKGDTVTIVVRE
AS121	SMKEEIKRLAEELKEKTKNEEIKRLAEEAAELAERSDDPEVLEVVKKALEEALKSKNEEKIELLLLVAVLVAEAGSVDAVEEKLEVALEALKAAEESKDPKLIRVALRLAIYALRQGLSAEAVRTALEIARLLAEEGVSEKLILALAKLAMYAVERGLPPEEVLERVRRLLEEVRRLGPQEAVLAYIEFAVEMMGVTMETHRSGNEVKVVIKGLHEELQEVLLELVLAVAEERGVRVRIRFKGDTVTIVVRE
AS122	SMKEEIKRLAEELKEKTKNEEIKRLAEEAAELAERSDDPEVLEVVKKALEEALKSKNEEKIELLLLVAVLVAEAGSVDAVEEKLEIALLALKKAEESKDPEVIRAALRIAIYALRQGLSAEAAKVALELAEELAERGYSEEVIIGLAEVALFALKRGKSPEEVKRKVLEAVERMEEAEPQEAVLIAIELAVELMGVTMETHRSGNEVKVVIKGLHEEEQEALLRLVLAVAEREGVRVRIRFKGDTVTIVVRE
AS123	SMKEEIKRLAEELKEKTKNEEIKRLAEEAAELAERSDDPEVLEVVKKALEEALKSKNEEKIELLLLVAVLVAEAGSVDAVEDKLEAALYALKVAEESKDPLVIRSALRIAIEALRVGASAEQVKKAIEIIKKAVEEGYPPEVVRTLETLLILALVVGKSVEELEKIYEELKKAYELESPQEAVLKVIEIAVKTMGVTMETHRSGNEVKVVIKGLHEMQQEYLLRAVLRAAEAWGVRVRIRFKGDTVTIVVRE

AS124	SMKEEIKRLAEELKEKTKNEEIKRLAEEAAELAERSDDPEVLEVVKKALEEALKSKNEEKIE LLLLVAVLVAEAGSVDAVEDKLEIALYALKVAEESKDPEKIRAALRLAIYALRIGADAELVKEAI EELIKVIEKIKDPKFIEVAELLLEAALYGGVKPEEIKKMIEELIEGYEKLSPQEFVLKVIELLVK AMGVTMETHRSGNEVKVVIKGLHEKQQELLLKAVLKAAELMGVRVRIRFKGDTVTIVVRE
AS125	SMKEEIKRLAEELKEKTKNEEIKRLAEEAAELAERSDDPEVLEVVKKALEEALKSKNEEKIE LLLLVAVLVAEAGSVDAVEDKLEIALTALKVAEESKDPEVIRLALRLAIEALRLGLDAEQVKTA LRLLKEAAEEGVDPEELRILALLLLLAALGGLPPEEMEEMVREYLEARRELGPQEFVLKAIE LAVKAMGVTMETHRSGNEVKVVIKGLHEEQQEALLRLVLETAELEGVRVRIRFKGDTVTIV VRE
AS126	SMKEEIKRLAEELKEKTKNEEIKRLAEEAAELAERSDDPEVLEVVKKALEEALKSKNEEKIE LLLLVAVLVAEAGSVDAVEKKLEVALEALKIAEESKDPEVIRAALRLAIEALRLGVVEHAVEAI KEIAKIIEEEKLSPEEIEVLIGLAILGLLRGLPAEEIKKMLKELAKILKGEKTQEAVLKVIELAVK AMGVTMETHRSGNEVKVVIKGLHEMQQELLLRIVLRTAEIMGVRVRIRFKGDTVTIVVRE
AS127	SMKEEIKRLAEELKEKTKNEEIKRLAEEAAELAERSDDPEVLEVVKKALEEALKSKNEEKIE LLLLVAVLVAEAGSVDAVEGKLEVALEALKIAEESKDPLVIRAALRIAIEALRHLEDFSRAPEH IRIAAEIVKEVKDPKEAEILVNIAILGLLAGLPVEELKKLLEEAKEILEKRKGQEEVLKIIEIAVK AMGVTMETHRSGNEVKVVIKGLHEHLQEILLRIVLTAAELMGVRVRIRFKGDTVTIVVRE
AS128	SMKEEIKRLAEELKEKTKNEEIKRLAEEAAELAERSDDPEVLEVVKKALEEALKSKNEEKIE LLLLVAVLVAEAGSVDAVEEKLEVALEALKFAEESKDPELIRAALRLAIEALRKGVDGKAVAE ALKKVKKLKEEGIPLKTLLLVLELLIALVKAGVTDKEAILAAIEELVEAQDLPPQELVLKVIELL VKAMGVTMETHRSGNEVKVVIKGLHEHQQEALLRAVLRAAEIEGVRVRIRFKGDTVTIVVR E
AS129	SMKEEIKRLAEELKEKTKNEEIKRLAEEAAELAERSDDPEVLEVVKKALEEALKSKNEEKIE LLLLVAVLVAEAGSVDAVEEKLEVALEALKLAEESKDPEIIRLALRVAIETLRTGGDAKIVAEA LKEVRELAEAGADPKTIRLMLRMMEALVRLGKVEKIKEAMERVREAAEAGLPPQELVLRVI EVAVEAMGVTMETHRSGNEVKVVIKGLHEHQQEALLRLVLGAAEIEGVRVRIRFKGDTVTI VVRE
AS130	SMKEEIKRLAEELKEKTKNEEIKRLAEEAAELAERSDDPEVLEVVKKALEEALKSKNEEKIE LLLLVAVLVAEAGSVDAVEEKLEAALAALKLAEESKDPEIIRGALRLAIEALRTGADGKTVVE VLKEVKKLYEEGELKAARLALELAIAAVRVGADGKVVLEEIKRLKKLYKEGAPPQELVLAVIE GAVKIMGVTMETHRSGNEVKVVIKGLHEEQQEALLRVVLKAAEIEGVRVRIRFKGDTVTIV VRE
AS131	SMKEEIKRLAEELKEKTKNEEIKRLAEEAAELAERSDDPEVLEVVKKALEEALKSKNEEKIE LLLLVAVLVAEAGSVDAVEHKLELALAALKVAEESKDPEKIRAALRLAITALREDVDAELAREI VEILEKLEEEEVSVEKFVLIAELLRLAVRVGADAERVREAVERLEEVFELESPQERVLGVIEI AVELMGVTMETHRSGNEVKVVIKGLHEEEQEALLRLVLKAAELEGVRVRIRFKGDTVTIVV RE
AS132	SMKEEIKRLAEELKEKTKNEEIKRLAEEAAELAERSDDPEVLEVVKKALEEALKSKNEEKIE LLLLVAVLVAEAGSVDAVEDKLEIALAALKLAEESKDPEIIRAALRFAIEALRVGAKAETVKKIL EIVKKAAEEGVDPRVIAAAVEAAAELLKVGVKGETAVKFVEEALEAAKLGDPQEAVLKVIEA LVKAMGVTMETHRSGNEVKVVIKGLHEEQQEALLRTVLRFAEIFGVRVRIRFKGDTVTIVV RE
AS133	SMKEEIKRLAEELKEKTKNEEIKRLAEEAAELAERSDDPEVLEVVKKALEEALKSKNEEKIE LLLLVAVLVAEAGSVDAVEGKLEAALIALKFAEESKDPEKIREALRLAITLLRVDVPEEEAPEL IKEALKAMEELKKPEDIELLGTLAIELLEVGVPAEKIKEALKEALEVFKLGDPQEQVLKIIEIM

	VKAMGVTMETHRSGNEVKVVIKGLHEKQQEALLRVVLKAAEKYGVRVRIRFKGDTVTIVVRE
AS134	SMKEEIKRLAEELKEKTKNEEIKRLAEEAAELAERSDDPEVLEVVKKALEEALKSKNEEKIELLLLVAVLVAEAGSVDAVEEKLEVALIALKEAEESKDPEVIRAALRLAIEMLRTGVADPEALKEVLKELKELVEEGKREAARIAAALAIALARVGRVEEIPEAVKELKEAIEKGLSPQEIVLKTIELMVKAMGVTMETHRSGNEVKVVIKGLHERQQEALLEVVLKTAEVTGVRVRIRFKGDTVTIVVRE
AS135	SMKEEIKRLAEELKEKTKNEEIKRLAEEAAELAERSDDPEVLEVVKKALEEALKSKNEEKIELLLLVAVLVAEAGSVDAVEEKLEIALEALKAAEESKDPELIRAALRIAIEALRTGAEAEVVKIALKELKEAAEEGFEPEALRVLGTFAIELIRLGVEAETLKEALKELKEVLKKGKTQEEVLKVIEIAVKAMGVTMETHRSGNEVKVVIKGLHEEQQEALLRIVLEAAEYTGVRVRIRFKGDTVTIVVRE
AS136	SMKEEIKRLAEELKEKTKNEEIKRLAEEAAELAERSDDPEVLEVVKKALEEALKSKNEEKIELLLLVAVLVAEAGSVDAVEGKLEIALEALKIAEESKDPEKIRIALRLAIEALREGVPAEAVKEILKEIEELYEEGDKEKARIAAELAIELIRVGVDAEAIVEAVKRLIEKLKEAEKPQEKVLIAIEEMVEAMGVTMETHRSGNEVKVVIKGLHEHQQEALLRLVLKVAEIEGVRVRIRFKGDTVTIVVRE
AS137	SMKEEIKRLAEELKEKTKNEEIKRLAEEAAELAERSDDPEVLEVVKKALEEALKSKNEEKIELLLLVAVLVAEAGSVDAVEEKLEIALEALKLAEESKDPELIRAALRIAIEALRTGADAEAVREALERLERLAEEGKKELLRLAAVIAIEAMRVGADAEALREALERLERAYEEGVPPQELVLTTIEEMVKLMGVTMETHRSGNEVKVVIKGLHEMQQEALLRVVLEAAELLGVRVRIRFKGDTVTIVVRE
AS138	SMKEEIKRLAEELKEKTKNEEIKRLAEEAAELAERSDDPEVLEVVKKALEEALKSKNEEKIELLLLVAVLVAEAGSVDAVEKKLEVALEALKIAEESKDPEIIRYALRLAIEALRRDVDAEKVIEALKELKKIAEEGLPPEILRIAGRLAIELVRVGKEEKVVEAIKKLVEAAKEGLPPQELVLKLIELMVEAMGVTMETHRSGNEVKVVIKGLHEEQQEALLEAVLYAAEVLGVRVRIRFKGDTVTIVVRE
H2	DEEVQEAVERAEELREEAEELIKKARKTGDPELLRKALEALEEAVRAVEEAICRNPDNDEAVETAVRLARELKKVAEELQERAKKTGDPELLKLALRALEVAVRAVELAIKSNPDNDEAVETAVRLARELKKVAEELQERAKKTGDPELLKLALRALEVAVRAVELAIKRNPDNEEAIKTALRLARELRKVAKELIERARKTGDAELLKKALEAARVAVEAVRLAAEYNKENAEKMAELLVELAELARLVADVLIELAEKTGDPELLKKALEVLEEAVEAVRLAIEYDPDHDEAVETAVRLARELKKVAEELQERAKKTGDPELLKLALRALEVAVRAVELAIKSNPDNEEAVETAKRLAEELRKVAELLEERAKETGDPCLQELAKRAKEVADRARELAKKSNPNN
E2-Target	SILELAEEVAEEIKEAIRIAKIGIARGLALELVKKEKTQEAVLKAIELFVRALGVTMETHRSGNEVKVVIKGLNIEEQVALYRLVREVSKLAGVETEIEVEGDTQTIVVRE
AS1-LgBiT	SMKEEIKRLAEELKEKTKNEEIKRLAEEAAELAERSDDPEVLEVVKKALEEALKSKNEEKIELLLLVAVLVAEAGSVDAVEEKLEIALLALKLAEESKDPRIIRGALRAAIAALRSDDPLALKTVKEALERARASKDERLIRAILAAAYAFALLAVAGASAERLKEAEAIVKELIAAAEKGASPQELVLLVIEMMVKGMGVTMETHRSGNEVKVVIKGLHESQQEVLLEAVLFAAELMGVRVRIRFKGDTVTIVVREGSSGSGGSGGGGSGGSSSGGVFTLEDFVGDWEQTAAYNLDQVLEQGGVSSLLQNLAVSVTPIQRIVRSGENALKIDIHVIIPYEGLSADQMAQIEEVFKVVYPVDDHHFKVILPYGTLVIDGVTPNMLNYFGRPYEGIAVFDGKKITVTGTLWNGNKIIDERLITPDGSMLFRVTINS
Target-SmBiT	EEAVRRRFEELLREALAFRERTGGRRETLEHAVRLARELAEFAASHPEFNRQEAVLLAIELMVRAMGVTMETHRSGNEVKVVIKGLNIDEQVALYRAVRETSKIMGVETEIEVEGDTQTIVVREGSSGSGGSGGGGSGGSSSGGVTGYRLFEEIL

SmBiTgraft	EVTGYRLFEEILETAKKLIEKLAKEE
AScov	EMKEEIRRLAEELRERTKDPEVRELAREAARLAEESDDEEVKEVVKKALEAALKSKDEEVIRLLLLAAVLAAAAARSGSPEEVLEVAKKALEAAMSSKDENVIRLALEIAVLAARANNKEVAELAKKVLELVLEAEKNPEKKEEVLEELIKLTEEVAESLPEEIREKILEALEALKEGKKEWTLQLLYELMRLADELGMAEISMVLSDAIYLFMKGVPEEVLIADLTVRLALVLAEK
LgBiT-SmBiTgraft-AS0	VFTLEDFVGDWEQTAAYNLDQVLEQGGVSSLLQNLAVSVTPIQRIVRSGENALKIDIHVIIPYEGLSADQMAQIEEVFKVVYPVDDHHFKVILPYGTLVIDGVTPNMLNYFGRPYEGIAVFDGKKITVTGTLWNGNKIIDERLITPDGSMLFRVTINSGGSGSGGSGGGGSGGSSSGGEVTGYRLFEEILETAKKLIEKLAKEEGGSGGSGGSGGGSEMKEEIRRLAEELRERTKDEEVRELAREAARLAEESDDEEVKEVVKKALEAALKSKDEEVIRLLLLAAVLAAAAARSGSPEEKLEIAKKALELAMKSKDERVIRGALKAAVAAARSDDELALKTVKEALEKAMASKDERLIRAILAAAYAFALLAVAGASAERLKEAEAIVKELIAAAEKGASPQELVLLVIEMMVKGMGVTMETHRSGNEVKVVIKGLHESQQEVLLEAVLFAAELMGVRVRIRFKGDTVTIVVRE
LgBiT-SmBiTgraft-AScov	VFTLEDFVGDWEQTAAYNLDQVLEQGGVSSLLQNLAVSVTPIQRIVRSGENALKIDIHVIIPYEGLSADQMAQIEEVFKVVYPVDDHHFKVILPYGTLVIDGVTPNMLNYFGRPYEGIAVFDGKKITVTGTLWNGNKIIDERLITPDGSMLFRVTINSGGSGSGGSGGGGSGGSSSGGEVTGYRLFEEILETAKKLIEKLAKEEGGSGGSGGSGGGSEMKEEIRRLAEELRERTKDEEVRELAREAARLAEESDDEEVKEVVKKALEAALKSKDEEVIRLLLLAAVLAAAAARSGSPEEVLEVAKKALEAAMSSKDENVIRLALEIAVLAARANNKEVAELAKKVLELVLEAEKNPEKKEEVLEELIKLTEEVAESLPEEIREKILEALEALKEGKKEWTLQLLYELMRLADELGMAEISMVLSDAIYLFMKGVPEEVLIADLTVRLALVLAEK
ASNeo2	SMKEEIKRLAEELKEKTKNEEIKRLAEEAAELAERSDDPEVLEVVKKALEEALKSKNLEKIELLLLVAVLVAEAGSVDAVEKKLEIALKALKFAEKSKDPEKIRLALRIAIILLRFEAETEEEEEELLKKFEEVLKLLEKGDYEKAVYLFIKTIFALLFKKRDPNDKELAEKIVDLIKERVEKEKGDIKKKIQLHAEHALYDALMILNIVKTNSPPAEEKLEDYAFNFYLILMEIAALFLKEGQYEEALKAVEMMVDMENIKTTEDEEEQERLANAIITILQSWIFS
ASNeo2 _cp03	MKKKIQLHAEHALYDALMILNIVKEGPGGSGGSGGSGGSGGSGSGPELEELKEEIKRLAEELKEKTKNEEIKRLAEEAAELAERSDDPEVLEVVKKALEEALKSKNLEKIELLLLVAVLVAEAGSVDAVEKKLEIALKALKFAEKSKDPEKIRLALRIAIILLRLEPETEEEEEEELLKVFEEVLKLLEKGDYEKAVKLFIEKIFELVKKRVEKVAEEKGNEKLRDYAFNFLLILEEIAKLFIEEGQYEEALKAVEMAVDMLNIMTTEDEEEQERLANAIITILQSWIFS
ASNeo2 _cp08	MKKKIQLHAEHALYDALMILNIVKEGPGGSGGSGGSGGSGGSGSGPEIEELKEEIKRLAEELKEKTKNPEIKRLAEEAAELAERSDDPEVLEVVKKALEEALKSKNLEKIELLLLVAVLVAEAGSVDAVEGKLKIALTALKAAEKSKNPEIIRIALRLAIAILRMEGLSEEELEELAEELEKMVEALEKGDKEEAKEAFKKILEKVKEKAIKNLEELKVDEKLYDYAFNFLLILEEIAKLFLEEGQYEEAVKAFEMMVYMDLIIKTKDEEEQEKLANAIITILQSWIFS
ASNeo2 _cp12	MKKKIQLHAEHALYDALMILNIVKEGPGGSGGSGGSGGSGGSGSGPELKELKEEIKRLAEELKEKTKNPEIKRLAEEAAELAERSDDPEVLEVVKKALEEALKSKNLEKIELLLLVAVLVAEAGSVDAVEGKLKIALTALKAAEKSKNPEIIRIALRLAIAILRLEGLSEEELEKIAELLEKMVEALEKGDKEEAKEAFEKILKIVVENVKKRLEELKKDEKLYDYAFNFILILEEIAKLFLEEGQYEEAVKALEMIVYMDLIIKTKDEEEQEKLANAIITILQSWIFS
ASNeo2_00	SMKEEIKRLAEELKEKTKDPEIIRLAEEAAELAERSDDPEVLEVVKKALEEALKSKNPEKIELLLLVAVLVAEAGSVDAVEEKLKIALYALKIAELSKNPELIRIALRAAIATLRNGNPAVIEEILKVLKEFAEKLEKGEEVTTKEFVEALIKATAEALAKLYPERKEEFLKLPELLKERVKKSKEDVKKKIQLHAEHALYDALMILNIVKTNSPPAEEKLEDYAFNFFLILFEIAYLFIKEEQYEEALKAIEMILY

	MERIKTLASEEEQEEFANAIITILQSWIFS
ASNeo2_01	SMKEEIKRLAEELKEKTKDPEIIRLAEEAAELAERSDDPEVLEVVKKALEEALKSKNPEKIEL<br>LLLVAVLVAEAGSVDAVEEKLEIALSALKIAELSKDPELIRVVLRAAIATLRNKNPAVLEKMRE<br>VLEKIAKKLEKGEKVTVEEVVESLLEATAKAMGEIMPDRKDEFEKLPEELKKKVKESNEDY<br>KKKIQLHAEHALYDALMILNIVKTNSPPAEEKLEDYAFNFYLILFEIAVLFLKEKQYEEALKAI<br>EMILYMEAIKYHASEEEQEEYANAIITILQSWIFS
ASNeo2_02	SMKEEIKRLAEELKEKTKNPEIKRLAEEAAELAERSDDPEVLEVVKKALEEALKSKNPEKIE<br>LLLLVAVLVAEAGSVDAVEEKLKIALIALKVAEESKDPLVIRYALRTAISYLRLKVSKEDEEEIV<br>KGVEKSAKIIEEEKDLKKAILTDLLVALKIAFKELGREDLEDVEGLIKKLLERAKKNKKDLKKK<br>IQLHAEHALYDALMILNIVKTNSPPAEEKLEDYAFNFVLILTEIAILFIVEGQYEEALKAIEMILD<br>MVQIKKTKDEEEQEEIANAIITILQSWIFS
ASNeo2_03	SMKEEIKRLAEELKEKTKNEEIIRLAEEAAELAERSDDPEVLEVVKKALEEALKSKNPEKIEL<br>LLLVAVLVAEAGSVDAVEEKLKVALAALKLAEESKDPETIRIALRLAIAVLRLKVPESAEEELE<br>KLAKVLEKALKEKMSTEELIKELTEVVLRLVAAALGFEEGEKLVEFLNKKIEEYKKNKKDIKK<br>KIQLHAEHALYDALMILNIVKTNSPPAEEKLEDYAFNFVLILTEIIALFLAEKQYEEALKAIEML<br>LRMAQIKLGASEEEQEELANAIITILQSWIFS
ASNeo2_05	SMKEEIKRLAEELKEKTRNEEIKRLAEEAAELAERSDDPEVLEVVKKALEEALKSKNPEKIE<br>LLLLVAVLVAEAGSVDAVEPKLKIALEALKFAEKSKDPEKIRLALRMAIILLRFKPKSKEEEEE<br>LLEAFKKVLELLEKGQYKEAVKLFMKTIFALLFKSKNPEDEELAKKIFEKILERAKKYKGDIK<br>KKIQLHAEHALYDALMILNIVKTNSPPAEEKLEDYAFNFYLILMEIAALFLKEGQYEEALKAV<br>EMMVLMENIKTVEDEEEQEKLANAIITILQSWIFS
ASNeo2_06	SMKEEIKRLAEELKEKTKNEEIKRLAEEAAELAERSDDPEVLEVVKKALEEALKSKNLEKIE<br>LLLLVAVLVAEAGSVDAVEGKLEVALEALKVAEESKDPEKIRIALRIAIYALRYKGSKEIEEKLL<br>EGTKEILEKLREGMPLEEAVLEYIKLVFIAALMELGYSEEEAEKIFEKMVERVKKIPESEIKK<br>KIQLHAEHALYDALMILNIVKTNSPPAEEKLEDYAFNFVLILYEIALLFIKLKQYEEALKAAEMI<br>LDMLNIKRTESEEEQEKLANAIITILQSWIFS
ASNeo2_07	SMKEEIKRLAEELKEKTKNEEIKRLAEEAAELAERSDDPEVLEVVKKALEEALKSKNPEKIE<br>LLLLVAVLVAEAGSVDAVEPKLRVALEALKVAEESKDPEKIRLALRIAIAVLRYKGKEELVEEL<br>AEKAVEILEKLREGAELKEVVKEYVKAVFVIALKELGYSEEEAEKIFEKLEERVKSLPKEEY<br>KKKIQLHAEHALYDALMILNIVKTNSPPAEEKLEDYAFNFVLILYEIALLFIELGQYEEAIKAAE<br>MILDMLNIKNAKTEEEQEEIANAIITILQSWIFS
ASNeo2_08	SMKEEIKRLAEELKEKTKNEEIKRLAEEAAELAERSDDPEVLEVVKKALEEALKSKNPEKIE<br>LLLLVAVLVAEAGSVDAVEAKLKVALAALKVAEESKNPEIIRLALRFAIALLRLKLPEEVEKKV<br>AEEAVKVLEKAKESGNPKELVLGFMEVLKVLFEEEGKEEEAYAVDYFLSKIEEYKKLSEEEI<br>KKKIQLHAEHALYDALMILNIVKTNSPPAEEKLEDYAFNFVLILLEIAMLFIALGQYEEAEKALI<br>MILDMKRIKESEDEEEQEEIANAIITILQSWIFS
ASNeo2_09	SMKEEIKRLAEELKEKTKNPEIIRLAEEAAELAERSDDPEVLEVVKKALEEALKSKNKEKIEL<br>LLLVAVLVAEAGSVDAVEPKLKIALAALKVAEESKNPEVIRLALRFAISLLRLELPEEVEEKVA<br>EAAVKALEEALEEEDPEELVKRFMEVIAVIFEELGEEEKARAIKYTLEKIKEYEKLSEEELKK<br>KIQLHAEHALYDALMILNIVKTNSPPAEEKLEDYAFNFVLILSEISALFIALKQYEEALKALEMI<br>LDMYRIKYEKDEKKQEEAANAIITILQSWIFS
ASNeo2_10	SMKEEIKRLAEELKEKTKNEEIKRLAEEAAELAERSDDPEVLEVVKKALEEALKSKNPEKIE<br>LLLLVAVLVAEAGSVDAVEKKLELALEALKVAEKSKNPEVIRLSLRLAISLLRLKAPEEAEEEL<br>LETAKKAIKALEEGKYEEFKKELIRLGAILLAILVGKLEDAEWLAEKYLERYEKLKKSKEDIKK

	KIQLHAEHALYDALMILNIVKTNSPPAEEKLEDYAFNFGLILIEIAILFLVEGQYEEALKAVEMIGRMYEIKTTESEEEQEELANAIITILQSWIFS
ASNeo2_11	SMKEEIKRLAEELKEKTRDEEIKRLAEEAAELAERSDDPEVLEVVKKALEEALKSKNKEKIELLLLVAVLVAEAGSVDAVEEKLEIALEALKYAEKEKDPELIRTVLRIAIGMLRLKVSKEEAEYLKETFLKALKEAEKEKDPEEKIATLILYSMVGALKLLDPEVAEPLLEKLKEKIEKLKKEKIDLKKKIQLHAEHALYDALMILNIVKTNSPPAEEKLEDYAFNFLLILMEIAVLFLAMKQYDEALKAVEMAFRMINIKNNASEEEQEEDANAIITILQSWIFS
ASNeo2_12	SMKEEIKRLAEELKEKTDNPEIQRLAEEAAELAERSDDPEVLEVVKKALEEALKSKNKEKIELLLLVAVLVAEAGSVDAVEPKLEAALKALKFAEKSKDPEMIRLALRAAILVLRYQPPPELQKELAEGLIKALEKAEKSGDAKVLVAEFNRHVASLAAKYIDPEMGELMKERFEELLKKLPEDKENIKKKIQLHAEHALYDALMILNIVKTNSPPAEEKLEDYAFNFILILSEIAVLFISEGQYEEALKASEMILAMLEIKETTSEKKQEEAANAIITILQSWIFS
ASNeo2_13	SMKEEIKRLAEELKEKTKDEEIKRLAEEAAELAERSDDPEVLEVVKKALEEALKSKNKEKIELLLLVAVLVAEAGSVDAVEPKLKVALAALKFAEKEKNPELIRAALRAAIAFLRLDLPPELEEEAAELLVKAIEEAEKEKNPEEVFKILVKTMIAVAALAFDPELAEKAREYFDKLVEKYKKEPGDLKKKIQLHAEHALYDALMILNIVKTNSPPAEEKLEDYAFNFFLILIEIAALFLVEGQYEEALKAAEMLLDMVRIKTTESEEEQEELANAIITILQSWIFS
ASNeo2_14	SMKEEIKRLAEELKEKTKNEEIIRLAEEAAELAERSDDPEVLEVVKKALEEALKSKNKEKIELLLLVAVLVAEAGSVDAVEPKLKIALEALKVAEESKNPKVIRFSLRAAIIALRNENLTEEEAEKIVELLKELLKAEKEGDEEKVKELIKEIFKVLAKSASGDETLKEAIDIIRKRVEEDKKNGVDLKKKIQLHAEHALYDALMILNIVKTNSPPAEEKLEDYAFNFKLILLEIAELFLELGQYEEALKAIDMIIRMDEIKKTKDEKEQERLANAIITILQSWIFS
ASNeo2_15	SMKEEIKRLAEELKEKTKNEEIKRLAEEAAELAERSDDPEVLEVVKKALEEALKSKNLEKIELLLLVAVLVAEAGSVDAVEGKLKVALEALKIAEESKDPLVIRASLRLAIAVLRLKGLSKESLEKITELLKKAAEALENKDTEGFLKALSEMVATALKDAGYEETAEAILKLTEKLLEEKENFNIDIKKKIQLHAEHALYDALMILNIVKTNSPPAEEKLEDYAFNFALILTEIAYLFALEGQYEEALKAFLMILRMLEIKQSEDEEEQEELANAIITILQSWIFS
ASNeo2_16	SMKEEIKRLAEELKEKTKNEEIKRLAEEAAELAERSDDPEVLEVVKKALEEALKSKNPEKIELLLLVAVLVAEAGSVDAVEGKLKVALEALKIAEESKNPEIIRIALRLAIAVLRQPNLSEEQLEEIKEQLKKFIEALEKGDIEEALKSLMKIFAILAEVALNQPHMKETMEKIIEEALKLKEEKVDFKKKIQLHAEHALYDALMILNIVKTNSPPAEEKLEDYAFNFSLILGEIAALFVLEGQYLEAVKALEMLVYMDLIKQDVSEEEQEKYANAIITILQSWIFS
ASNeo2_17	SMKEEIKRLAEELKEKTKNPEIKRLAEEAAELAERSDDPEVLEVVKKALEEALKSKNLEKIELLLLVAVLVAEAGSVDAVEGKLKIALTALKAAEKSKNPEIIRIALRLAIAILRFEDLSEEELEKIAEALEKMVEALEKGDKEEAIESFLEIMAILLKTILKDESMKEILKKILERAKKEKEEGKDLKKKIQLHAEHALYDALMILNIVKTNSPPAEEKLEDYAFNFYLILAEIAVLFLLEGQYEEAVKAFEMIVYMDLIKKTKDEEEQEKLANAIITILQSWIFS
ASNeo2_18	SMKEEIKRLAEELKEKTKNEEIKRLAEEAAELAERSDDPEVLEVVKKALEEALKSKNPEKIELLLLVAVLVAEAGSVDAVEEKLKIALNALKIAEISKNPELIRTALRLAIAILRADLPKELLEKAVEIFEKALKMLEEGKDPKEVIKYLIEGSLVVALMAFGFKEEEAKEYLKKLLEKAKKLTEEEIKKKIQLHAEHALYDALMILNIVKTNSPPAEEKLEDYAFNFMLILAEIAALFIASGQYEEATKAVEMMFYMYAIKTSASEEEQEEFANAIITILQSWIFS
ASNeo2_19	SMKEEIKRLAEELKEKTKNEEIKRLAEEAAELAERSDDPEVLEVVKKALEEALKSKNPEKIELLLLVAVLVAEAGSVDAVEKKLKIALAALKIAEKSKNPELIRTALRLAIAFLRVDAPEELEELAVEIFEEALEMLEKGENEEEIIEKLIKGSLIIALMLYGYEREEAEKILEELLKKAKELSEEEAKKKI

	QLHAEHALYDALMILNIVKTNSPPAEEKLEDYAFNFMLILAEIAALFISLKQYEEAEKAVEMMFYMYAIKTSASEEEQEEFANAIITILQSWIFS
ASNeo2_20	SMKEEIKRLAEELKEKTKDPEIKRLAEEAAELAERSDDPEVLEVVKKALEEALKSKNPEKIELLLLVAVLVAEAGSVDAVEGKLKIALTALKVAEESKNPWLIRLALRTAIELLRLPHITEEQAEAIVKLLEGLLKAEKEGNKEEVKKLLKELFKELAAIATNDESMKKYFDEALEKAKKEKEEGVDLKKKIQLHAEHALYDALMILNIVKTNSPPAEEKLEDYAFNFHLILIEIAALFIVEGQYEEAEKAILMMLRMEAIKTTEDEELQERYANAIITILQSWIFS
ASNeo2_21	SMKEEIKRLAEELKEKTKNEEIKRLAEEAAELAERSDDPEVLEVVKKALEEALKSKNEEKIELLLLVAVLVAEAGSVDAVEGKLKIALTALKVAEESKNPFLIRLSLRIAIGVLRISNITEEQAEKIVKLLEELLKAEKEGNEEKFEELFKELAATLAEIASGDERMKEYFYEALEKAKKWKEEGVDLKKKIQLHAEHALYDALMILNIVKTNSPPAEEKLEDYAFNFHLILIEIAALFIVEKQYEEAAKAMLMMLDMKVIKESENEELQEKLANAIITILQSWIFS
ASNeo2_22	SMKEEIKRLAEELKEKTKNEEIKRLAEEAAELAERSDDPEVLEVVKKALEEALKSKNEEKIELLLLVAVLVAEAGSVDAVEEKLKVALKALKIAEKSKDPEVIRISLRLAISFLRLKPPEEEEEKVAEEILKALEYLEKGLTKENVKEALKELVKAVMILLGLPEEAEKMAEEFEKYIEELEKKDIDLKKKIQLHAEHALYDALMILNIVKTNSPPAEEKLEDYAFNFALILLEIAALFLVEGQYEEAFKAYEMVFLMEAIKTSESEEEQEKAANAIITILQSWIFS
ASNeo2_23	SMKEEIKRLAEELKEKTKDPEIKRLAEEAAELAERSDDPEVLEVVKKALEEALKSKNPEKIELLLLVAVLVAEAGSVDAVEGKLKVALAALKVAEESKNPELIRTALRLAIALLRLDLPEEVEEKVSEAAVKALEKAREEGSLEVLVKEFMEVLAKLYEELGKKEEAEFIKKELEKIEEYKKLPEEEIKKKIQLHAEHALYDALMILNIVKTNSPPAEEKLEDYAFNFVLILSEISALFMALGQYEEAAKALEMILYMEYIKTSASEEEQEKLANAIITILQSWIFS

References

1. Dietz, H., Berkemeier, F., Bertz, M. & Rief, M. Anisotropic deformation response of single protein molecules. *Proc. Natl. Acad. Sci.* **103**, 12724–12728 (2006).

2. West, D. K., Brockwell, D. J., Olmsted, P. D., Radford, S. E. & Paci, E. Mechanical Resistance of Proteins Explained Using Simple Molecular Models. *Biophys. J.* **90**, 287–297 (2006).

3. Quijano-Rubio, A. *et al.* A split, conditionally active mimetic of IL-2 reduces the toxicity of systemic cytokine therapy. *Nat. Biotechnol.* **41**, 532–540 (2023).

4. Moal, I. H. & Fernández-Recio, J. SKEMPI: a Structural Kinetic and Energetic database of Mutant Protein Interactions and its use in empirical models. *Bioinformatics* **28**, 2600–2607 (2012).

5. Schreiber, G., Haran, G. & Zhou, H.-X. Fundamental Aspects of Protein−Protein Association Kinetics. *Chem. Rev.* **109**, 839–860 (2009).

6. Selzer, T., Albeck, S. & Schreiber, G. Rational design of faster associating and tighter binding protein complexes. *Nat. Struct. Biol.* **7**, 537–541 (2000).

7. Miyashita, O., Onuchic, J. N. & Wolynes, P. G. Nonlinear elasticity, proteinquakes, and the energy landscapes of functional transitions in proteins. *Proc. Natl. Acad. Sci.* **100**, 12570–12575 (2003).
