# Supplementary material for: Design of facilitated dissociation enables timing of cytokine signalling
Source: Nature. 2025 Sep 24;647(8089):528–35. doi: 10.1038/s41586-025-09549-z (PMC12611780; doi:10.1038/s41586-025-09549-z)
Supplement: Supplementary file 2 — Reporting Summary [file 41586_2025_9549_MOESM2_ESM.pdf]

Corresponding author(s): David Baker, Adam Broerman, Florian Praetorius

Last updated by author(s): 08/06/2025

Reporting Summary

Nature Portfolio wishes to improve the reproducibility of the work that we publish. This form provides structure for consistency and transparency in reporting. For further information on Nature Portfolio policies, see our Editorial Policies and the Editorial Policy Checklist.

Statistics

For all statistical analyses, confirm that the following items are present in the figure legend, table legend, main text, or Methods section.

n/a	Confirmed	
☐	☒	The exact sample size (n) for each experimental group/condition, given as a discrete number and unit of measurement
☐	☒	A statement on whether measurements were taken from distinct samples or whether the same sample was measured repeatedly
☐	☒	The statistical test(s) used AND whether they are one- or two-sided<br>*Only common tests should be described solely by name; describe more complex techniques in the Methods section.*
☒	☐	A description of all covariates tested
☒	☐	A description of any assumptions or corrections, such as tests of normality and adjustment for multiple comparisons
☐	☒	A full description of the statistical parameters including central tendency (e.g. means) or other basic estimates (e.g. regression coefficient) AND variation (e.g. standard deviation) or associated estimates of uncertainty (e.g. confidence intervals)
☐	☒	For null hypothesis testing, the test statistic (e.g. F, t, r) with confidence intervals, effect sizes, degrees of freedom and P value noted<br>*Give P values as exact values whenever suitable.*
☒	☐	For Bayesian analysis, information on the choice of priors and Markov chain Monte Carlo settings
☒	☐	For hierarchical and complex designs, identification of the appropriate level for tests and full reporting of outcomes
☐	☒	Estimates of effect sizes (e.g. Cohen's d, Pearson's r), indicating how they were calculated

Our web collection on statistics for biologists contains articles on many of the points above.

Software and code

Policy information about availability of computer code

Data collection	RFdiffusion (https://github.com/RosettaCommons/RFdiffusion), inpainting with RosettaFold (https://github.com/RosettaCommons/RFDesign), ProteinMPNN (https://github.com/dauparas/ProteinMPNN), and AlphaFold2 were used along with custom code to design proteins, which has been deposited at Zenodo (https://doi.org/10.5281/zenodo.16749263). PyMOL (v2.5.5) was used during some protein design steps. Pulse shapes for DEER experiments were calculated with PulseShape (https://github.com/StollLab/PulseShape). MD simulations were performed using GROMACS 2020.2. SPR data was collected on a Biacore 8k with Biacore Insight software version 5.0.18.22405. FP, FRET, and luminescence data was collected on a Synergy Neo2 plate reader with BioTek Gen5 software version 3.11 or 3.14.
Data analysis	Proteins were visualized with PyMOL (v2.5.5). Kinetic data from SPR, FP, FRET, and luminescence measurements was analyzed with custom code. Nonlinear least squares optimization was performed with LMFIT version 1.3.1. Crystallography data was analyzed using CCP4 program suite (v8.0, particularly the modules Pointless, Aimless, Phaser, and Coot v0.9.8), Phenix (v1.21.1), MolProbity (https://github.com/rlabduke/MolProbity/tree/641e0eb52e88fc5f4b99409f297b82020620177c), and XDS (20230630). DEER data was analyzed using eprTools (https://github.com/mtessmer/eprTools). MD data was analyzed with GROMACS 2020.2 and MDAnalysis version 2.4.3. Single molecule co-tracking was analyzed using SLIMfast4C available at https://zenodo.org/record/5712332. The Matlab R2022b script for FRET efficiency analysis accompanied with a demo dataset is provided as Supplementary Software in https://doi.org/10.1038/s41467-024-49876-9. Flow cytometry data was analyzed with CytExpert (v2) or FlowJo (v10.10.0). Statistics were performed using GraphPad Prism (v10.5.0). RNA-seq data was analyzed with Rsubread (v2.16.1, particularly with the module featureCounts after alignment), DESeq2 (v1.42.1), and FGSEA (v1.28.0). Code to analyze all data and to generate all figures has been deposited at Zenodo (https://doi.org/10.5281/zenodo.16749448).

For manuscripts utilizing custom algorithms or software that are central to the research but not yet described in published literature, software must be made available to editors and reviewers. We strongly encourage code deposition in a community repository (e.g. GitHub). See the Nature Portfolio guidelines for submitting code & software for further information.

Data

Policy information about availability of data

All manuscripts must include a data availability statement. This statement should provide the following information, where applicable:

- Accession codes, unique identifiers, or web links for publicly available datasets
- A description of any restrictions on data availability
- For clinical datasets or third party data, please ensure that the statement adheres to our policy

All data generated during this study are included in either the main text or as Supplementary Information. Data deposition, atomic coordinates, and structure factors for all crystal structures reported in this paper have been deposited in the Protein Data Bank (PDB), https://www.rcsb.org/ with accession codes 9DCX, 9DCY, 9DCZ, 9DD0, 9DD1, 9DD2, 9DD3, 9DD4, 9DD5, and 9OLQ. PDB models and sequences for all designs and source data with analysis scripts has been deposited at Zenodo under https://doi.org/10.5281/zenodo.16749448. Single molecule tracking data has been deposited at Zenodo: calibration beads, unstimulated samples, long term measurements and labeled ligand experiments under https://doi.org/10.5281/zenodo.13957447, Neo2 and Neo2 + effector under https://doi.org/10.5281/zenodo.13957498 and ASNeo2 and ASNeo2 + effector, as well as smFRET data under https://doi.org/10.5281/zenodo.13957540. The raw RNA-seq data have been deposited in the NCBI Sequence Read Archive (SRA) under BioProject accession code PRJNA1302552 at https://ncbi.nlm.nih.gov/bioproject/PRJNA1302552. The SKEMPI database can be accessed at https://life.bsc.es/pid/skempi2/database/index. The reference genome GRCh38 (accession code GCF_000001405.26) can be accessed at https://hgdownload.soe.ucsc.edu/goldenPath/hg38/bigZips/.

Research involving human participants, their data, or biological material

Policy information about studies with human participants or human data. See also policy information about sex, gender (identity/presentation), and sexual orientation and race, ethnicity and racism.

Reporting on sex and gender	Sex and gender information was not collected in this study.
Reporting on race, ethnicity, or other socially relevant groupings	Race and ethnicity information was not collected in this study.
Population characteristics	This study included no human participants.
Recruitment	This study included no human participants.
Ethics oversight	This study included no human participants requiring ethics oversight.

Note that full information on the approval of the study protocol must also be provided in the manuscript.

Field-specific reporting

Please select the one below that is the best fit for your research. If you are not sure, read the appropriate sections before making your selection.

☒ Life sciences ☐ Behavioural & social sciences ☐ Ecological, evolutionary & environmental sciences

For a reference copy of the document with all sections, see nature.com/documents/nr-reporting-summary-flat.pdf

Life sciences study design

All studies must disclose on these points even when the disclosure is negative.

Sample size	Sample sizes were chosen to obtain confident enough measurements to demonstrate significant differences between groups. The main text SPR, CD, FP, FRET, and luminescence experiments, and also a subset of DEER and SEC experiments, were performed multiple times, and one representative sample was reported due to low variance between replicates. Having established low variance for these experiments, similar experiments in the supplement were performed multiple times or once. All microscopy, cell staining, and qPCR/RNA-seq measurements were conducted with sample size >=3. No sample size calculations were performed; rather the sample sizes were chosen based on experience and were sufficient for the important observed differences between groups to be strongly statistically significant.
Data exclusions	In Fig 5h, we excluded from analysis signaling activity data obtained from batches of YT-1 cells that were unresponsive to stimulation.
Replication	All main text SPR, CD, FP, FRET, and luminescence measurements were performed multiple times to ensure reproducibility and low variance. Having established low variance for these experiments, similar measurements in the supplement were performed multiple times or once. To establish reproducibility of SEC experiments in the supplement, designs AS0, AS1, AS2, AS5, AS116, AS117, and AS118 were tested 3 times from 3 separate expressions with similar results, so other SEC experiments were performed once. A subset of DEER experiments were performed multiple times with similar results, so other DEER experiments were performed once. In particular, SPR in Fig. 2d,h, 4b; FRET in Fig. 4c; SEC in Fig. S9a were performed twice. SPR in Fig. 2a,e,f,g; FP in Fig. 4b, S5c, S12; luminescence in Fig. 4d,e; SEC in Fig. S6b; DEER with AS1 in Extended Data Fig. 2 were performed three or more times. For all microscopy, cell staining, and qPCR/RNA-seq experiments, three or four independent biological replicates were performed unless otherwise noted in the figure caption. All attempted replications of all experiments were successful, with the exception that some batches of YT-1 cells were unresponsive to stimulation; the findings in Fig. 5h were not observed with those batches.

Randomization: Allocation was random.

Blinding: Blinding was not relevant because all groups in each experiment were analyzed the same way.

Reporting for specific materials, systems and methods

We require information from authors about some types of materials, experimental systems and methods used in many studies. Here, indicate whether each material, system or method listed is relevant to your study. If you are not sure if a list item applies to your research, read the appropriate section before selecting a response.

Materials & experimental systems

n/a	Involved in the study
☐	☒ Antibodies
☐	☒ Eukaryotic cell lines
☒	☐ Palaeontology and archaeology
☒	☐ Animals and other organisms
☒	☐ Clinical data
☒	☐ Dual use research of concern
☒	☐ Plants

Methods

n/a	Involved in the study
☒	☐ ChIP-seq
☐	☒ Flow cytometry
☒	☐ MRI-based neuroimaging

Antibodies

Antibodies used: Alexa Fluor® 647-conjugated Phospho-STAT5 (Tyr694) rabbit monoclonal antibody (Cell Signaling Technology, #9365, C71E5), nanobodies for Single-molecule tracking experiments: Anti-GFP and anti-ALFAtag (produced in-house).
The following antibodies were purchased from BioXCell: human CD3 (OKT3, BE0001-2) and human CD28 (9.3, BE0248). The following antibodies or staining reagents were purchased from BioLegend: human CD3 (OKT3, 317324), human GATA3 (W19195B, 386906), human CD69 (FN50, 310932), human CD25 (BC96, 302611), human BCL2 (100, 658708), human Ki-67 (Ki-67, 350526), and Human TruStain FcX™ (422302). The following antibodies were purchased from BD Biosciences: pSTAT5 (47/Stat5, 612599), and BD Pharmingen™ FITC Active Caspase-3 Apoptosis Kit (571606).

Validation: Surface marker antibodies purchased from BioLegend (human CD3 (OKT3, 317324), human CD69 (FN50, 310932), human CD25 (BC96, 302611)) were validated by the manufacturer: "Each lot of this antibody is quality control tested by immunofluorescent staining with flow cytometric analysis (https://www.biolegend.com/protocols/cell-surface-flow-cytometry-staining-protocol/4283/)."

BioLegend human GATA3 antibody (W19195B, 386906) was validated by the manufacturer: "Each lot of this antibody is quality control tested by intracellular flow cytometry using our True-Nuclear™ Transcription Factor Staining Protocol."

BioLegend human BCL2 antibody (100, 658708) was validated by the manufacturer: "Each lot of this antibody is quality control tested by intracellular immunofluorescent staining with flow cytometric analysis (https://www.biolegend.com/protocols/intracellular-flow-cytometry-staining-protocol/4260/)."

BioLegend human Ki-67 antibody (Ki-67, 350526) was validated by the manufacturer: "Each lot of this antibody is quality control tested by our Ki-67 staining protocol (https://www.biolegend.com/en-gb/products/pe-cyanine7-anti-human-ki-67-antibody-9084)."

BD Biosciences pSTAT5 antibody (47/Stat5, 612599) was validated by the manufacturer: "This purified or conjugated mAb was characterized by flow cytometry" yielding positive staining for human PBMC and whole blood cells treated with IL-2.

BD Pharmingen™ FITC Active Caspase-3 Apoptosis Kit (571606) was validated by the manufacturer by flow cytometry.

Cell Signaling Technology pSTAT5 antibody (Tyr694, C71E5, #9365) was validated by the manufacturer: "This Cell Signaling Technology antibody is conjugated to Alexa Fluor® 647 fluorescent dye and tested in-house for direct flow cytometric analysis of human cells."

Antibodies purchased from BioXCell (human CD3 (OKT3, BE0001-2) and human CD28 (9.3, BE0248)) were not validated for binding by the manufacturer, but we found they stimulated the proliferation of human T cells as expected.

Eukaryotic cell lines

Policy information about cell lines and Sex and Gender in Research

Cell line source(s): HeLa cells for single molecule imaging: German Collection of Microorganisms and Cell Cultures GmbH (ACC 57), YT (CD25+) cells: American Type Culture Collection (ATCC) previously used in [Gaggero et al., Science Immunol 2022, PMID 36459543]

Authentication: HeLa cells were authenticated by the manufacturer: "STR analysis according to the global standard ANSI/ATCC ASN-0002.1-2021 (2021) resulted in an authentic STR profile of the reference STR database." YT cells were authenticated by Eurofins using STR profiling.

Mycoplasma contamination	HeLa cells were tested negatively for mycoplasma (PCR).
Commonly misidentified lines (See ICLAC register)	No commonly misidentified cell lines were used in this study.

Plants

Seed stocks	*Report on the source of all seed stocks or other plant material used. If applicable, state the seed stock centre and catalogue number. If plant specimens were collected from the field, describe the collection location, date and sampling procedures.*
Novel plant genotypes	*Describe the methods by which all novel plant genotypes were produced. This includes those generated by transgenic approaches, gene editing, chemical/radiation-based mutagenesis and hybridization. For transgenic lines, describe the transformation method, the number of independent lines analyzed and the generation upon which experiments were performed. For gene-edited lines, describe the editor used, the endogenous sequence targeted for editing, the targeting guide RNA sequence (if applicable) and how the editor was applied.*
Authentication	*Describe any authentication procedures for each seed stock used or novel genotype generated. Describe any experiments used to assess the effect of a mutation and, where applicable, how potential secondary effects (e.g. second site T-DNA insertions, mosiacism, off-target gene editing) were examined.*

Flow Cytometry

Plots

Confirm that:

☒ The axis labels state the marker and fluorochrome used (e.g. CD4-FITC).

☒ The axis scales are clearly visible. Include numbers along axes only for bottom left plot of group (a 'group' is an analysis of identical markers).

☒ All plots are contour plots with outliers or pseudocolor plots.

☒ A numerical value for number of cells or percentage (with statistics) is provided.

Methodology

Sample preparation	For the YT signaling assay (Fig. 5h), cells were stimulated with 1 nM ASNeo2 or Neo2 for 5 minutes at 37°C and then either immediately fixed in 4% PFA at 37°C or transferred to a new well containing Effector, Ruxolitinib or nothing. Cells were then fixed in 4% PFA at 37°C at different timepoints and finally re-fixed in 4% PFA for 15 minutes at room temperature. Following fixation, the cells were washed once with PBS containing 0.5% BSA (PBSA) and permeabilized with 100% methanol for 45 minutes at 4°C. After permeabilization, the cells were washed twice with PBSA and stained for 1 hour at room temperature using Alexa Fluor® 647-conjugated Phospho-STAT5 (Tyr694) rabbit monoclonal antibody (Cell Signaling Technology, #9365). The cells were washed three times, then analyzed by flow cytometry.<br>For T cell surface marker staining (Ext. Data Fig. 9e), cells were collected into U-bottom 96-well plates (Thermal Fischer Scientific), blocked with Human TruStain FcX™ (BioLegend), and incubated with indicated antibodies at 4 °C for 20 min, followed by live/dead staining by 4',6-diamidino-2-phenylindole (DAPI, Thermo Fisher Scientific). Cells were then washed and resuspended with FACS buffer (PBS containing 0.2 % BSA, Sigma-Aldrich) for flow cytometry analyses. For T cell phospho-STAT staining (Ext. Data Fig. 9h), primary human T cells were rested in T cell medium lacking IL-2 for 24h before signaling assays. Cells were plated in a 96-well round bottom plate in 50µl T cell medium. Cells were stimulated with 50 µL of ASNeo2 for 5 or 25 minutes, followed by the addition of effector peptide and incubation for another 20 minutes at 37°C, and the reaction was terminated by fixation with 1.5% paraformaldehyde (PFA) for 15min at room temperature with agitation. Cells were washed and permeabilized with ice-cold 100% methanol for 60min on ice. Afterward, cells were washed with FACS buffer before staining with pSTAT5 antibodies for 1h at 4°C in the dark. Cells were washed and resuspended in FACS buffer for flow cytometry analyses. For T cell caspase-3 staining (Ext. Data Fig. 9g), cells were first stained for surface markers and Zombie Violet Fixable Dye (BioLegend), followed by staining with a FITC Active Caspase-3 Apoptosis Kit (BD Biosciences) according to the manufacturer's protocol. For transcription factor staining, cells were first stained for surface markers and Zombie Violet Fixable Dye, then fixed and permeabilized using a Foxp3/Transcription Factor Staining Buffer Set (eBioscience) as per the manufacturer's instructions. Cells were subsequently incubated with the indicated antibodies for intracellular staining. Detection was performed using a CytoFlex (Beckman Coulter), and data were analyzed with FlowJo (v10.10.0).
Instrument	CytoFlex S flow cytometer (Beckman Coulter)
Software	CytExpert, FlowJo
Cell population abundance	For the YT signaling assay, no cell sorting was performed. For the primary T cell assays, the proportion of $CD3^+$ T cells among live singlets ranged from 90% to 100%.
Gating strategy	YT cell populations were identified based on forward/side scatter profiles. Primary T cells were gated sequentially on singlets, live cells, and then $CD3^+$ cells. Gating examples for YT cells and primary T cells are shown in Supp. Fig. 17.

☒ Tick this box to confirm that a figure exemplifying the gating strategy is provided in the Supplementary Information.
